# Predicting Parasite Dynamics in Mixed-Use Trans-Himalayan Pastures to Underpin Management of Cross-Transmission Between Livestock and Bharal

**DOI:** 10.3389/fvets.2021.714241

**Published:** 2021-09-29

**Authors:** Munib Khanyari, Kulbhushansingh R. Suryawanshi, E. J. Milner-Gulland, Eleanor Dickinson, Abhirup Khara, Rashmi Singh Rana, Hannah Rose Vineer, Eric R. Morgan

**Affiliations:** ^1^Nature Conservation Foundation, Bangalore, India; ^2^Interdisciplinary Centre for Conservation Sciences, Oxford, United Kingdom; ^3^Department of Biological Sciences, University of Bristol, Bristol, United Kingdom; ^4^Snow Leopard Trust, Seattle, WA, United States; ^5^School of Biological Sciences, Queen's University, Belfast, United Kingdom

**Keywords:** disease, Trans-Himalaya, livestock, nematodes, bharal, model, ungulates, grazing

## Abstract

The complexities of multi-use landscapes require sophisticated approaches to addressing disease transmission risks. We explored gastro-intestinal nematode (GINs) infections in the North India Trans-Himalayas through a socio-ecological lens, integrating parasite transmission modelling with field surveys and local knowledge, and evaluated the likely effectiveness of potential interventions. Bharal (blue sheep; *Pseudois nayaur*), a native wild herbivore, and livestock share pasture year-round and livestock commonly show signs of GINs infection. While both wild and domestic ungulates had GINs infections, egg counts indicated significantly higher parasite burdens in bharal than livestock. However, due to higher livestock densities, they contributed more to the total count of eggs and infective larvae on pasture. Herders also reported health issues in their sheep and goats consistent with parasite infections. Model simulations suggested that pasture infectivity in this system is governed by historical pasture use and gradually accumulated larval development during the summer, with no distinct short-term flashpoints for transmission. The most effective intervention was consequently predicted to be early-season parasite suppression in livestock using temperature in spring as a cue. A 1-month pause in egg output from livestock could lead to a reduction in total annual availability of infective larvae on pasture of 76%, potentially benefitting the health of both livestock and bharal. Modelling suggested that climate change over the past 33 years has led to no overall change in GINs transmission potential, but an increase in the relative influence of temperature over precipitation in driving pasture infectivity. Our study provides a transferable multi-pronged approach to investigating disease transmission, in order to support herders' livelihoods and conserve wild ungulates.

## Introduction

Globally, land conversion and intensification of land use means that wildlife habitats and livestock pastures increasingly overlap, creating more intensive, multi-use landscapes (1). A factor driving this intensification of contact is the increasing demand for livestock products. Although much of this global demand is met by intensive livestock farming, there are c.752 million low-income livestock herders (earning < $2/day) who carry out extensive herding on rangelands where wildlife is also present (2, 3). This leads to the potential for disease cross-transmission, which can impact on both the incomes of resource-poor herders (4) and wildlife conservation (5).

**Figure 1:**
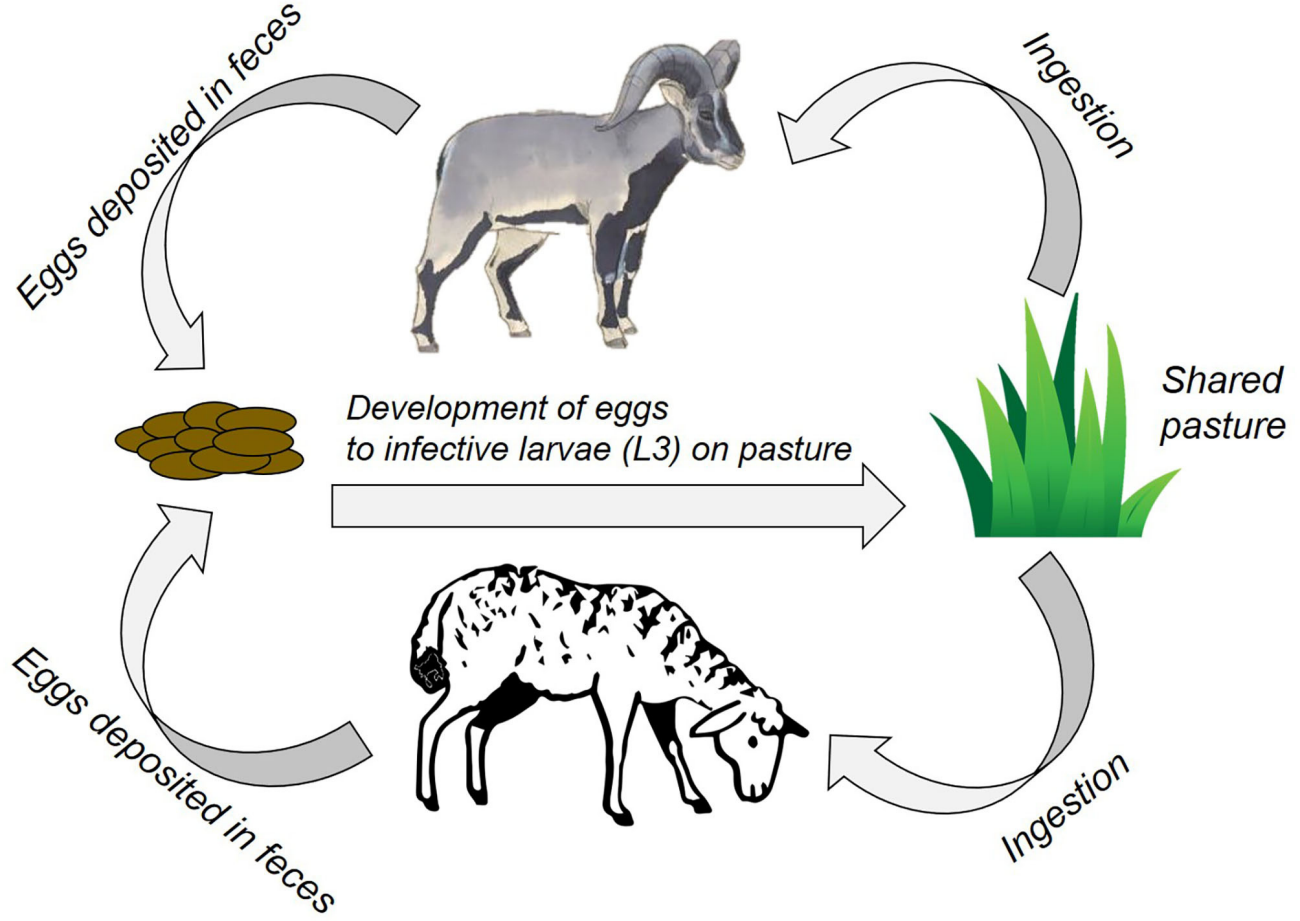
A schematic for GIN transmission in our case study site. Above: Bharal, Below: Livestock (sheep/goat). Sheep icon courtesy Francesco Cesqo Stefanini (noun project) and bharal courtesy Sartaj Ghuman.

Amongst a diverse set of disease-causing agents, endoparasites (particularly gastro-intestinal nematodes, GINs) are important determinants of fitness in wild ungulates (6, 7). They also impact milk production, growth rates, fertility, and susceptibility to other diseases in livestock (8, 9), and are economically costly to farmers in both monetised and informal economies (10). Transmission is through indirect contact by sharing pasture and water points (11), leading to ingestion of free-living infective stages in the environment [(12); Figure 1]. Often GINs get overlooked in assessments of disease risks, as their impact can be subtle and clinical signs hard to detect, even though they can cause large aggregate impacts on health and productivity. Importantly, since part of GINs life history is driven by environmental conditions, climate change could alter their transmission in ways that are difficult to predict but which may have substantial impacts on both wildlife and livestock health (13).

Understanding impacts and transmission of GINs is especially difficult, yet pertinent, in remote and harsh multi-use landscapes, home to both wildlife and livestock-dependent herders. These landscapes often offer little access to veterinary facilities and advice (14), meaning knowledge of GIN impacts and implementation of control measures may be very limited. Most studies of these systems are limited to short time-scales, providing only a snapshot of current conditions, which may vary between years. This means that measuring important variables for understanding GINs transmission, such as infective larval density, is challenging (15). Given the difficulties and hazards of disease control interventions directed at wild species, GINs management is often focussed on livestock. Yet, crucially, trialling control options in practise is difficult, expensive (16) and can be damaging to the animals concerned (e.g., where treatment is withheld). Therefore, models can be useful to explore potential interventions in a virtual environment before attempting them in practise (5). While most GINs models are species-specific (17), the main GINs species in wildlife and livestock broadly share climatic envelopes and parameter values. Therefore, general livestock GINs models can be used to understand GINs transmission in multi-use landscapes (18). Additionally, people who share habitats with wildlife have first-hand experience of wildlife-livestock interactions (19). They too can provide rich information concerning the health of both livestock and wild ungulates (20).

The Indian Trans-Himalaya region is symbolic of remote multi-use landscapes. Most of the area is inhabited by agro-pastoral societies. This region also harbours a unique assemblage of mountain ungulates, which maintain vegetation structure and serve as the main prey for rare predators such as the Snow Leopard, *Panthera uncia*, and Tibetan wolf, *Canis lupus* (21, 22). Whilst there is significant literature examining the competition for resources between domestic and wild ungulates in these areas [e.g., (23)], disease dynamics has been subject to less research. Disease cross-transmission between wild and domestic ungulates is particularly likely in these settings because parasite host range often mirrors host phylogeny (24). These regions are particularly sensitive to changes in climate (25), which can have numerical, functional and micro-evolutionary impacts on parasite-host assemblages (13).

Despite the importance of GINs for livestock productivity and wildlife conservation, understanding of transmission risk, assessment of the potential effects of climate and climate change, and recommendations for appropriate interventions at the livestock-wildlife interface across multi-use landscapes are all still limited (26). More case studies are needed to understand these issues, particularly from temperate multi-use landscapes, as they remain understudied with respect to disease dynamics. High mountain areas can also serve as model systems to understand dynamics across other temperate areas with comparable land use and climatic patterns (27); and to understand shifts in species distribution under climate change in vertical as well as horizontal planes [e.g., (28)].

Due to the socio-ecological complexities of multi-use landscapes, a multi-pronged interdisciplinary approach is needed. With this in mind, we explored disease transmission risk between wildlife and livestock in the Kibber area of the Indian Trans-Himalaya through a socio-ecological lens and, based upon that, evaluated the likely outcomes of potential interventions. Specifically, we aimed to investigate the general dynamics of the multi-use system with respect to GINs parasite transmission and, by investigating climate cues to trigger interventions, explored what actions taken in livestock herds might limit cross-transmission to wild ungulates. We did so by collating existing datasets and collecting additional data to parameterise a GINs transmission model and then building on and contextualising the model using Traditional Ecological Knowledge (TEK) (19). We modelled bharal (blue sheep; *Pseudois nayaur*) because it is the only wild ungulate in the study area, and sheep and goats because they are most likely to share GINs with bharal (Figure 1). Our integrated approach could form a basis for discussions with local stakeholders on introducing locally-applicable and socially-relevant interventions to better align people's socio-economic priorities with wildlife conservation.

## Materials and Methods

### Study Area

The 186,000 km^2^ of the Indian Trans-Himalaya, includes parts of the Tibetan Plateau and its marginal mountains (29). Our study area included Kibber village and its surrounding livestock pasture, which is within Lahaul-Spiti district, Himachal Pradesh. The region is characterised by low precipitation (*c*.500 mm annually, with most precipitation in the form of winter snow), a short growing season, low primary productivity, and high livestock densities (29). This high-altitude (3,500–6,700 m) region experiences extreme climatic conditions, with winter temperatures ranging from −35 to 3°C, and summer temperatures ranging from 1 to 28°C. Our field study period was the entire year of 2018 (1st January-31st December).

The vegetation is classified as “Alpine scrub” or “dry Alpine steppe” (30). The large mammalian fauna includes bharal and their predators the snow leopard and the wolf. This region is also home to one herd of Asiatic Ibex *Capra sibirica* but they are spatially separated from the pasture shared by bharal and livestock, by a deep gorge. Agro-pastoralist communities, have inhabited this region for 2–3 millennia. The livestock assemblage includes sheep, goats, cattle, cattle-yak hybrids, yaks, donkeys and horses. Cattle, donkeys, cow-yak hybrids, goats, and sheep are herded to pasture (herded stock), while yaks and horses are free ranging. Herded stock are shepherded to the pastures every morning and brought back to stocking pens inside the village in the evening. Families take turn shepherding the entire village's herded stock alongside a designated village shepherd. Most families own small land holdings (c.1.5 ha) for cultivation, mainly of barley, *Hordeum vulgare*, for subsistence, and green pea, *Pisum sativum*, as a cash crop.

### Data Collection

**Figure 2:**
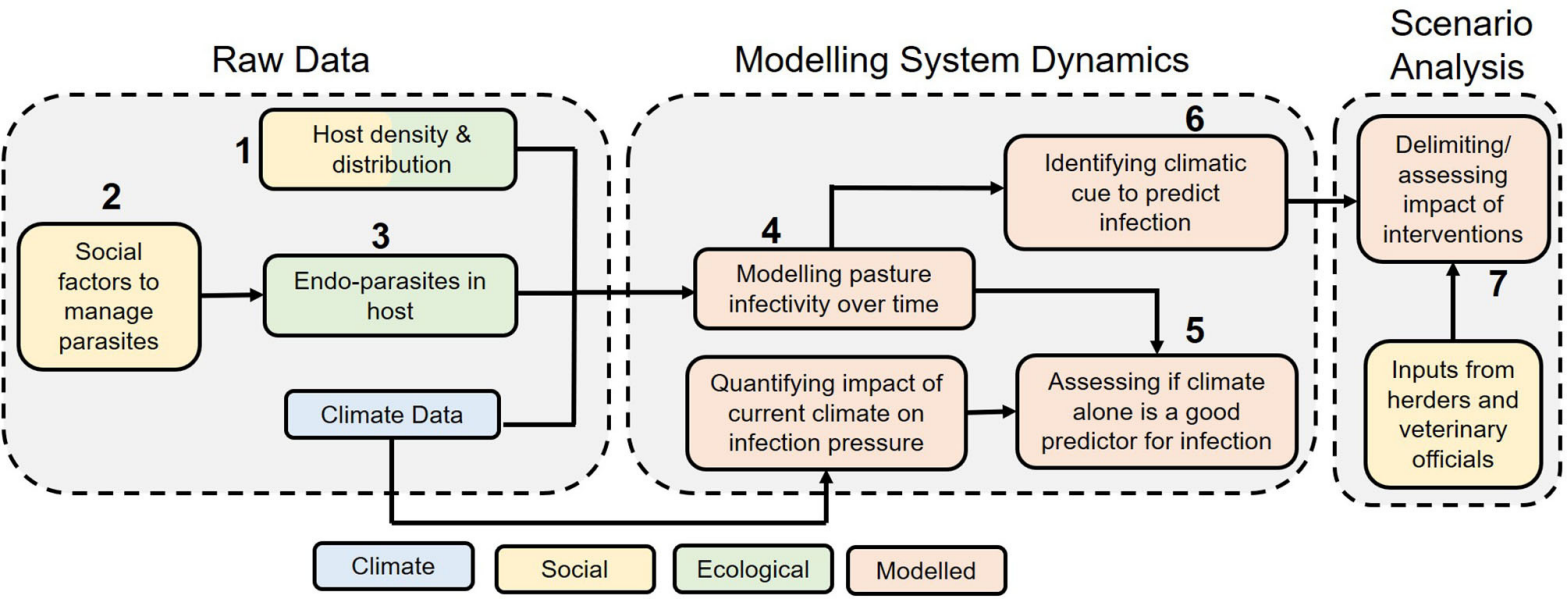
A schematic showcasing how the different forms of evidence combine to give an overall picture of the two-host (bharal, and sheep + goats combined) GIN system to inform control measures. The numbers are linked to numbers in Table 1 below.

Data collection aimed to assess the relationship between wild and domestic ungulates based on spatial overlap and potential GINs parasite cross-transmission. Further data on levels of nematode eggs in faeces over time were collected and used to calibrate a parasite transmission model (based on 17), which enabled evaluation of cross-transmission risk under different scenarios, including alternative management strategies. Figure 2 and Table 1 articulate how different forms of evidence were combined to investigate the dynamics of this GINs system in order to inform management.

**Table 1:** Research questions with reasoning and methods used to answer those questions, in order to understand two-host GIN system dynamics and inform measures using Kibber as a case study.

No.^*^	**Research Question**	**Method (section number)**	**Reasons**
1	*Do hosts share pasture?*	Focus group discussions and double-observer surveys (2.2.1, 2.2.2, and 2.3.1)	Contact patterns form the basis of GIN disease transmission (11), and are used as inputs to the GIN transmission model
2	*What management techniques exist for small ruminant health?*	Semi-structured interviews (2.2.3)	Social interventions can influence GIN presence in livestock and consequently transmission to wild ungulates (31)
3	*What is the abundance and diversity of endoparasites in hosts?*	Faecal egg counts (FEC) (2.2.4 and 2.3.2)	Presence of endoparasites can result in cross-transmission given appropriate contact patterns (11). Nematode FEC are used as inputs to the GIN transmission model
4.	*What is the magnitude and seasonality of pasture infectivity?*	GLOWORM-FL model (2.4.1 and 2.4.2)	The GLOWORM-FL tracks the number and density of infective larvae over time incorporating climate data driving the life history of nematode parasites
5.	*Is climate alone a good predictor of infection pressure?*	Relationship between GLOWORM-FL and *Q_0_* outputs (2.4.1, 2.4.2, and 2.4.4)	Climate alone can be considered a good predictor of infection pressure when a large proportion of variation in GLOWORM-FL output is explained by its simplified, purely climate-driven *Q_0_* formulation. Time and relative intensity of infections can be then determined by climatic variables alone to consequently inform interventions. This would bypass the effort needed to analyse faecal samples as inputs to GLOWORM-FL model, and to take detailed account of grazing patterns
6.	*Can we delimit climatic cues to predict infection pressure?*	(2.4.3)	Based on area under the curve (AUC) of infective larval (*L3*) abundance, as predicted by the GLOWORM-FL model, and further extracting the importance of rainfall to infection pressure (see text). Under low rainfall dependence, parasite management can be more simply guided by temperature profile
7.	*Can we delimit interventions and assess their impact?*	% reduction in intensity of infective larvae on pasture (2.4.5)	Based on the modelling outputs (4–6) and consulting with local stakeholders we defined potential interventions and predicted their impacts in reducing pasture infectivity

*Hosts refers to bharal and small ruminants (sheep + goats combined). GIN, gastro-intestinal nematode; FEC, faecal egg count; AUCL3_h_, area under the curve of predicted infective larval (L3_h_) density, a measure of overall nematode infection pressure. For explanation of GLOWORM-FL and Q_0_ models, and AUC arising from the models, see text.*

* *Corresponds to the numbers on Figure 2*.

#### Livestock Density and Distribution

We focused on small ruminants (sheep and goats combined), which are taxonomically closest to bharal and hence most likely to share parasite species (24). In the explanations below, therefore, livestock is taken to indicate sheep and goats combined. The abundance and distribution of livestock was estimated through focus group discussions (FGDs) and resource mapping with herders from Kibber (32). We conducted five FGDs, with 12, 8, 10, 6, and 7 people, respectively. Each group included animal owners and shepherds. The FGDs involved discussion of the livestock distribution in pastures surrounding Kibber across the year. Each group built a resource map displaying locations of areas grazed by livestock from Kibber. Discrepancies amongst the five maps produced were settled in discussion with the village headman. The boundaries were then delimited on Google Earth Pro. Livestock abundance in Kibber was obtained from the village headman through a key informant interview. This number was triangulated with interviews of herders in Kibber (see below). Total abundance of livestock was divided by the area of utilised pasture to obtain an estimate of average density.

#### Bharal Density and Distribution

**Figure 3:**
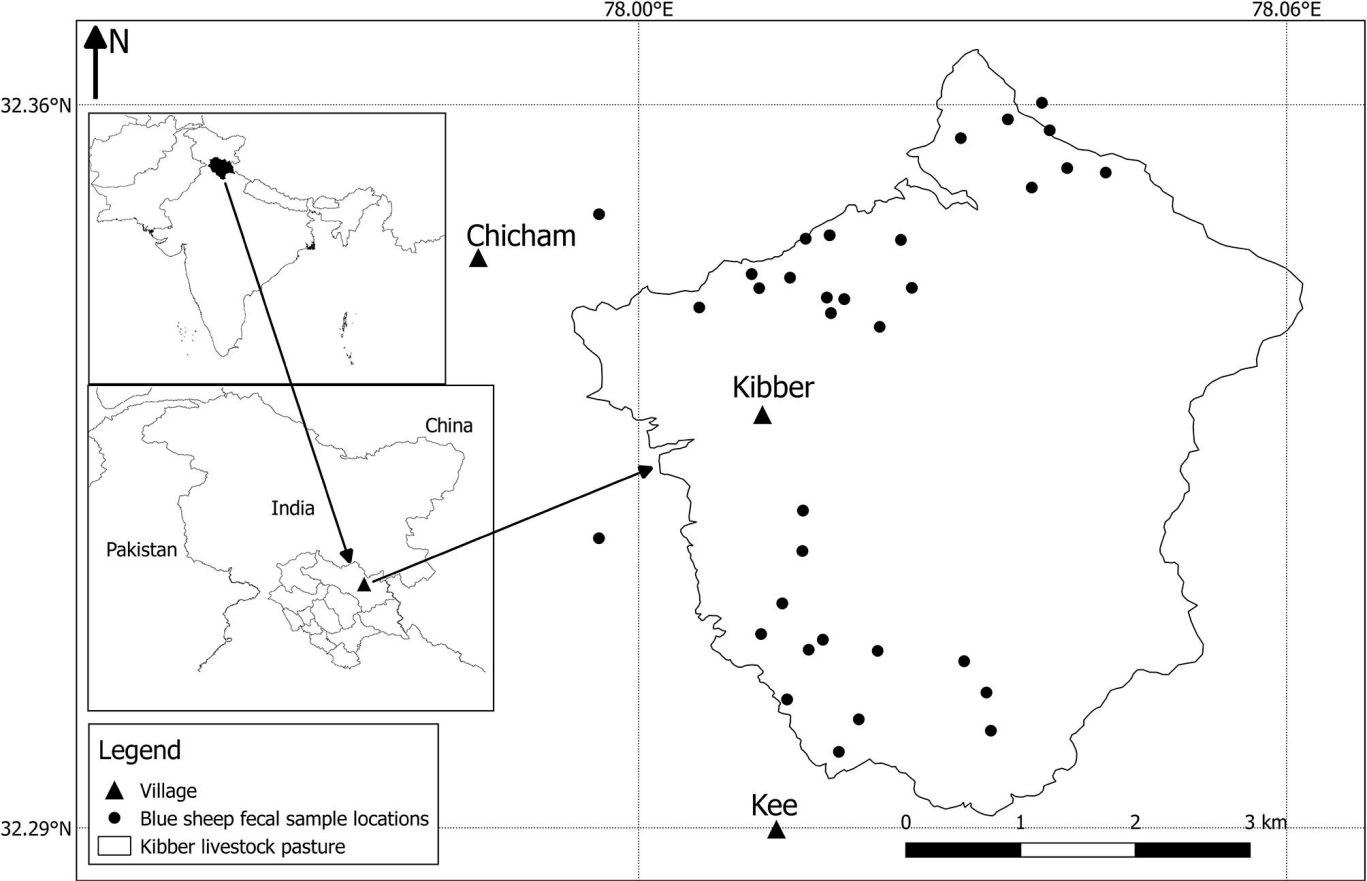
Map displaying bharal (blue sheep) faecal sample locations within the Kibber livestock pasture. The inset maps show the location of the study area within the Lahaul-Spiti district of Himachal Pradesh state, India.

We assessed bharal abundance in May 2018, using the double-observer method (33). This method uses two observers separated in time and space to count wild ungulates, and then estimates abundance within a mark-recapture modelling framework which controls for detection probability. The study area was defined as the livestock pasture surrounding Kibber (Livestock Density and Distribution; Figure 3). The area was divided into two blocks. Each block was surveyed for wild ungulates, with the aim of complying with the three main assumptions of the method: (i) entire visual coverage of the survey area can be achieved; (ii) the counts of the two observers are independent; and (iii) the two observers collect adequate information to be able to identify individual herds based on the age-sex composition, herd location (using a Global Positioning System device) and any other peculiarities. This is so that individual herds can be identified and the proportion spotted by both observers calculated. The population-specific data collected were group size and group detection or non-detection by both the observers.

Bharal are not known to undertake long-distance vertical or horizontal seasonal migrations. Several studies on bharal in Kibber have found them using similar pasture locations at different times of year, suggesting limited (if any) defined seasonal elevation migration (33–36). To validate this, we conducted an ungulate mapping exercise within our FGDs, asking where and when bharal were found within the pastures surrounding Kibber.

#### Management of Livestock Health

Semi-structured interviews (Supplementary Material 1) were conducted with 32 (57%) of the 56 livestock owners and herders in Kibber, to gain insights on livestock health and management practises. A local field assistant was present during interviews to help in translation. The interviews were conducted in Hindi, which is spoken well by most herders and the interviewers (MK and RS). The translator clarified doubts, if any, using the local Spitian dialect of Tibetan. The interview was designed to collect information about: i) general health of livestock, especially small ruminants, ii) prevalent endoparasites, and iii) livestock health management practises. Two key informant interviews were conducted with a government veterinarian with responsibility for the Spiti region and a veterinary assistant. To triangulate answers regarding endoparasite presence we conducted Five-Point Checks (37) and faecal analysis (see Endoparasite Burdens in Bharal and Livestock). The Five-Point Checks were conducted together by the livestock owners and authors (MK and RSR). Five-Point Checks provide an indication of the impact of endoparasites on host health, by scoring five signs of parasitism. The five elements consist of body condition (1 = fat to 5 = thin; reversed from original to maintain consistency of higher scores meaning poorer health), faecal breech soiling (1 = clean to 5 = dirty), anaemia (1 = red to 5 = pale), nasal discharge (0 = absent, 1 = present), and submandibular oedema or bottle jaw (0 = absent, 1 = present). Lower values are indicative of relatively healthy hosts with respect to parasitism (low impact of endoparasites), whilst higher numbers are indicative of relatively unhealthy ones with respect to parasitism (high impact of endoparasites). For simplicity, we reduced body condition, anaemia and faecal breech soiling scores to simple thresholds where the five points were converted to 0 (score <3; lacking signs of endo-parasitism) or 1 (score 3 or above; displaying signs of endo-parasitism). Total score for each individual was calculated as the sum of modified individual element scores, hence from minimum 0 (healthy) to maximum 5 (unhealthy); and then averaged across each livestock herd (*n* = 32) within Kibber. For the interviews and Five-point check data, we also bootstrapped responses with replacement (10,000 iterations) and estimated means and 95% confidence intervals. Non-overlapping confidence intervals were interpreted as being statistically significant.

All surveys involving people and the Five Point Checks were approved by the University of Bristol's Ethical Committee. Each respondent was told that their identity would be kept anonymous and that they would not be identified by name or specific location in any publications or other communication. Consent was taken orally before conducting the surveys and checks and all the responses were coded, with names or other identities not recorded to ensure anonymity.

#### Endoparasite Burdens in Bharal and Livestock

Fresh faecal pellet samples were collected from sheep, goats and bharal. Collection was opportunistic, with recently-voided faeces collected from the ground, and covered every month of the year. Faecal Egg Counts (FEC) were conducted on pooled faecal samples, to evaluate the density of helminth eggs and coccidial oocysts excreted onto the pasture to seed onward transmission, and as an indirect indicator of parasite burden. The mini-FLOTAC method (38) was used as a field-friendly, simple and cost-effective method for FEC in remote areas. This method estimates the abundance and diversity of endoparasites, using sedimentation-flotation to separate ova of nematodes and protists from the faecal matter and allow them to be identified morphologically and quantified under a microscope.

Livestock samples were pooled at the level of the overall Kibber livestock herd and bharal samples were pooled at the level of the study population (39). For bharal, samples were collected from all age-sex classes. The date and location of each sample collected was recorded (Supplementary Material 2). For the sheep and goats, which are herded as one unit, we collected fresh faecal samples, taking various samples from different individuals and trying to get as many as possible at a time. Subsequently, we placed all the individual samples, each of similar volume, together into a plastic bag and mashed and mixed them thoroughly using digital pressure. From the mixed composite (pooled) sample, we took 5 g faeces and mixed that thoroughly with 45 ml saturated sodium chloride salt solution, and then examined the suspension under a microscope at medium power, following the mini-FLOTAC method (38). The same procedure was followed for bharal. Sample hereafter refers to a pooled sample.

The number of eggs found for each parasite was recorded for each sample and multiplied by a factor of 5 to obtain the total FEC in eggs per gramme (EPG) of faeces. If multiple samples from the same host type were analysed on a given date (Supplementary Material 2), an average EPG was taken. We were particularly interested in the FEC of strongyle nematodes as they are used as input into the GLOWORM-FL model (see 2.4.2). Nevertheless, as there are limited data available on endoparasites from the Indian trans-Himalayas, all other endoparasites that were identified were quantified as well.

### Data Analysis

#### Bharal Abundance

The total number of bharal groups was estimated using the two-survey mark-recapture procedure in the Bayesian “BBRecapture” package of R (40, 41). Following Suryawanshi et al. (33), the analysis was conducted with groups as the unit. A group was coded “11” if recorded by both teams, “10” if only the first team recorded it, and “01” if only the second team recorded it. We modelled the probability of detection for the two teams separately (using the “mt” model in R package “BBRecapture”). Details on model fitting to estimate population size and confidence intervals are in Supplementary Material 3.

#### FEC Data Analysis

As parasite counts are typically over-dispersed, we used the non-parametric bootstrap *t*-test to compare the levels of faecal egg density between wild and domestic ungulates (42), running separate *t*-tests for each recognised type of parasite ovum. The strongyle nematode FEC, with the dates of collection and daily temperature and precipitation values, were further used as inputs into a model to predict levels of pasture infectivity over time, and therefore the potential for cross-transmission between domestic and wild ungulates (see Transmission Models).

### Transmission Models

#### Climate Data

Directly measured meteorological data were not available for Kibber, and so interpolated datasets were used to estimate parasite vital rates within the population dynamic models (below). Daily temperatures and precipitation were obtained from the POWER Data Access Viewer (DAV) which is made available by the National Aeronautics and Space Administration (NASA) (43). We used the POWER Single Point Data Access widget which provides access to near real-time 0.5 ×0.5 degree datasets by single point (lat/long). This was obtained for the years 1985–2018.

#### Transmission Model—Predicting Pasture Infectivity Over Time

The predictive model is based on the life cycle of the free-living stages of trichostrongylid GINs (Equations 1–7), as specified in the GLOWORM-FL model (17). The GLOWORM-FL model estimates the development of parasites from eggs, after they have been deposited by host, to the third-stage infective larvae (*L3*), and their translation onto pasture. This results in an estimate of the magnitude (number) of *L3* that are available for ingestion. The number of eggs per gramme of faeces (FEC) is multiplied by host faecal output (f) and the density of the host species (D) to estimate egg output on pasture (E) (Equation 1). Nematodes from overlapping cohorts are tracked, with new deposited eggs (E_new_) getting added to existing eggs, upon accounting for a moisture-limited development success correction factor (C) (Equation 2). The development of *L3* in faeces (*L3*_*f*_) from eggs (E), via the pre-infective larval stage (L), is subjected to temperature-dependent stage-specific mortality rates (μ_1_) and development rate (δ) (Equations 3, 4).

A climate-dependent (temperature and moisture) horizontal migration rate (m_1_) is used to estimate the migration of *L3* from faeces onto pasture (*L3*_*p*_). As *L3*_*p*_ can reside in either the herbage (*L3*_*h*_) or the soil (*L3*_*s*_), random bi-directional movement between soil and herbage is simulated with substrate specific mortality rates (μ_4_, μ_5_), and a temperature-dependent vertical migration rate (m_2_) (Equations 5–7).

We ran the model in R version 3.6.3 (41), using the *lsoda* function in the “deSolve” package (44). This makes use of an Adams-backward differentiation formulae (BDF) with an adaptive integration method. The model output is the daily number of individual GINs per hectare for each life-stage. The model predicts the density of *L3* on pasture from which we calculate *L3*_*h*_ per kg dry herbage (*L3*/kgDM) by dividing *L3*_*h*_ (Equation 7) by the biomass of dry herbage per hectare (parameterized from 44). Annual infection pressure (i.e., number of *L3* which animal can be exposed during the year) was obtained by area under the curve (AUC*L3*_*h*_), which was calculated by summing daily L*3*_*h*_ per kg dry herbage values. Peak infection (i.e., highest number of *L3* on herbage) day was obtained by calculating the mode of the model output (pilot analysis revealed distinct singular peaks). Lastly, to parameterize the model, FEC were used as input (see FEC Data Analysis) and host weights, averaged across sexes to account for sexual dimorphism (bharal and livestock) were obtained from literature [52 kg bharal—(45); 22.4 kg sheep/goat Spiti Livestock Husbandry Department 2018]. Faecal output was assumed to be the same for both species [7.0 g h^−1^ (46)].

Model output was used to indicate pasture infectivity, and calculate changes to infectivity as a result of climate and management (see sub-sections below). The model was run for three host scenarios: only bharal; only livestock; and bharal and livestock combined. In this way the relative contribution of each host to overall pasture infectivity was estimated. We used *Teladorsagia circumcincta* parameters, because this species tends to dominate over the warm-adapted *Haemonchus contortus* in cool temperate areas, and has similar responses to climate outside the host as other common genera such as *Trichostrongylus* (47). Additionally, studies from the western Himalayan regions (similar to Kibber) have found *Trichostrongylus* species to be common in small ruminants (48, 49). Additional details about the model can be found in Rose et al. (17) and the parameters used here are displayed in Supplementary Material 4.


(1) $$\begin{array}{l} E_{new}\frac{\;}{\;}=D\left(f\;\times FEC\right) \end{array}$$

(2) $$\begin{array}{l} \frac{dE}{dt}=\;-\left(\mu_{1}+2\delta \right)E+E_{new}C \end{array}$$

(3) $$\begin{array}{l} \frac{dL}{dt}=\;-\left(\mu_{2}+2\delta \right)L+2\delta E \end{array}$$

(4) $$\begin{array}{l} \frac{{dL3}_{f}}{dt}=\;-\left(\mu_{3}+m1\right){L3}_{f}+2\delta L \end{array}$$

(5) $$\begin{array}{l} \frac{{dL3}_{p}}{dt}=\;-\mu_{4}\left(\left(1-m_{2}\right){L3}_{p}\right)-\;\mu_{5}\left({m_{2}L3}_{p}\right)+m_{1}{L3}_{f} \end{array}$$

(6) $$\begin{array}{l} {L3}_{s}\frac{\;}{\;}=\;{L3}_{p}\left(1-m_{2}\right) \end{array}$$

(7) $$\begin{array}{l} {L3}_{h}\frac{\;}{\;}=\;{L3}_{p}m_{2} \end{array}$$

#### Identifying Climatic Drivers of Predicted Larval Abundance

Precipitation and temperature are both drivers of larval availability, but their relative importance varies according to prevailing climate. Effective intervention strategies and appropriate weather cues consequently differ between temperature-driven and precipitation-driven systems. We evaluated the relative importance of precipitation and temperature in Kibber by comparing model outputs under actually-observed precipitation, with theoretical values generated when precipitation is set to a high, non-limiting value. First, we calculated the area under the curve (AUC*L3*_*h*_) by summing the daily *L3*_*h*_ per kilogramme of herbage for each day over a given year. Then, we ran the model first using actual precipitation (for 2018) and then using constant high precipitation (c. 2.5 times higher than the highest daily precipitation value of 41.32 mm = 100 mm/day), which removes rainfall constraints on larval development and migration. The quotient $\frac{L_{3}AUC\;real\;precipitation}{L_{3}AUC\;high\;precipitation}\;$indicates the extent to which rainfall limits transmission. Values can vary from approaching zero (transmission is strongly limited by rainfall, since larval availability under real, observed, rainfall is much lower than it could potentially be under increased rainfall) to one (increasing rainfall to saturating levels does not increase larval availability, therefore real rainfall is not limiting). This calculation is based on moisture acting on the free-living stages based on minimum thresholds that permit development in faeces and migration onto herbage, such that above these thresholds additional rainfall no longer increases transmission. A high quotient indicates that nematode transmission is effectively driven solely by temperature, with rainfall rarely, if ever, limiting. The calculation therefore suggests which climatic cue would be most useful to trigger parasite control measures, e.g., after precipitation or based on warmth.

#### Quantifying the Contribution of Current Climate to Infection Pressure

The GLOWORM-FL model tracks the number and density of infective larvae over time, and can be condensed into a time-invariant formulation to estimate the potential for population growth under current epidemiological conditions, given additional assumptions regarding parasite lifespan and fecundity, and host density and feeding. The resulting basic reproduction quotient for parasites, *Q*_0_, is analogous to *R*_0_ for microparasites (50).

The *Q*_0_ model incorporates environmental conditions (temperature and precipitation) and host factors (density and herbage intake) to estimate the second generation mature worms produced by a single adult worm throughout its lifetime, in the absence of density-dependent constraints such as immunity and within-host competition. The value of *Q*_0_ is estimated by equation 8 (50):


(8) $$\begin{array}{l} Q_{0}=\frac{\text{q}\gamma }{\text{u}}\frac{\beta r}{\rho +\beta \text{H}\;}{\text{Hm}}_{2} \end{array}$$

Where, *Q*_0_is calculated from fecundity (γ), adult mortality (μ), ingestion of rate of *L3* by the host (β), mortality rate of *L3* on pasture (ρ), establishment rate of ingested *L3* (p), density of hosts (H) and a vertical larval migration parameter (m_2_). The parameter *q*, which describes the probability of an egg developing to *L3* and reaching herbage, was expanded (equation 9) as described by Rose et al. (50), thus incorporating climate dependence in the life history of free-living stages along with another, horizontal, migration parameter (m_1_). This allows for non-linear interactions between development (δ), survival (μ_i_) and horizontal migration rates.


(9) $$\begin{array}{l} q=\frac{\delta m_{1}}{\left(u_{e}+\;\delta \right)\left(u_{l3}+m_{1}\right)} \end{array}$$

As in the GLOWORM-FL model, *Q*_0_ was estimated using parameters for *Te. circumcinta* (Table 2). The “geosphere,” “deSolve,” and “forecast” packages in R were used to the run the model. Temperature and precipitation data were used as for the GLOWORM-FL model.

**Table 2:** Extended *Q*_0_ model parameter definitions and estimates, based on values for *Teladorsagia circumcincta*; and within-host stages in sheep.

**Parameter**	**Definition**	**Value**	**References**
**γ**	Fecundity (eggs day^−1^ per adult)	228.261	(51)
*u*	Instantaneous daily mortality rate of adult nematodes	0.0307	(52)
*q*	Probability that an egg will develop to *L3* and migrate onto pasture	$\frac{\delta \;m1}{\left(\mu_{e}\;+\;\delta \right)\left(\mu_{l3}\;+\;m1\right)},\frac{P}{E}\geq \;1$, $0,\;\frac{P}{E}\leq 1\;$	(17, 53)
**δ**	Instantaneous daily development rate of eggs to *L3*	−0.02085 +0.00467 T_mean_	(17)
	Instantenous daily mortality rate of eggs	Exp (−1.62026-0.17771*T+0.00629*T^2^)	(17)
	Instantenous daily mortality rate of *L3* in faeces	10*exp (−4.58817-0.13996T+0.00461*T^2^)	(17)
m_1_	Instantenous daily *L3* migration rate between faeces and pasture	0.21	(17)
ρ	Instantenous daily mortality rate of *L3* on pasture	μ_3_/3	(54)
m_2_	Proportion of total pasture *L3* that are found on herbage	0.2	(55, 56)
*r*	Probability of establishment of ingested *L3*	0.127	(57)
β	Rate of ingestion of *L3* on pasture	C/BA	–
*c*	Daily herbage dry matter intake per host (kg DM day1)	1.4	(52)
*H*	Host density or stocking density (sheep per ha)	This either varies regionally or held constant	(58)
*B*	Standing biomass (kg DM ha^−1^)	2,000 or taken regionally	(52)
*A*	Grazing area (ha)	1	–
P	Total daily precipitation (mm)	Daily variable	(43)
*E*	Daily potential evapotranspiration (mm day1)	0.0023 * 0.408 *R_*a*_($\frac{T_{\max}+\;T_{\min}}{2}+17.8)\;\sqrt{T_{\max}-T_{\min}}$	(59)
*R_*a*_*	Extra-terrestrial radiation (MJ m^−2^ day^−1^)	Daily variable	(60)
*T_*mean*_, T_*min*_, T_*max*_*	Mean, minimum and maximum daily temperature (°C)	Daily variable	(43)

An advantage of using *Q*_0_ rather than GLOWORM-FL is that historic information on pasture occupancy is not required; rather, it estimates the extent to which current conditions favour transmission. FEC data are also not required as input, unlike for the GLOWORM-FL model, since *Q*_0_ is scaled to the individual worm. This approach can successfully identify times and places of high transmission potential in the absence of detailed host information [e.g., (54)] and predict future spatial and seasonal patterns of transmission under climate change (50). It is likely to be more reliable when current climatic conditions have a dominant influence on transmission success, and less reliable when historical factors such as past climate and pasture occupancy are more important.

To investigate the extent to which short term climatic variation explains infection pressure in the Kibber system, we ran a Pearson's correlation test between the predicted values of *Q*_0_ in a given year, and the predicted total level of herbage contamination with *L3*_*h*_. To do so, daily *Q*_0_ and its area under the curve (AUC*Q*_0_), daily available *L3*_*h*_ per kilogramme of herbage calculated using the GLOWORM-FL model, and the area under that curve (AUC*L3*_*h*_) were calculated for each year between 1985 and 2018. AUC*L3*_*h*_ per kilogramme of herbage (infection) and AUC *Q*_0_ (reproductive rate) were then correlated for the period 1985–2018. We also calculated $\frac{L_{3}AUC\;real\;precipitation}{L_{3}AUC\;high\;precipitation}\;$for each of these years to investigate if the quotient changed over time. To check for autocorrelation between the time series data points, we plotted residuals of each time series model. Time series model output, built using the “ggscatter” package in R, was reported only if the residuals were uncorrelated and had zero mean.

#### Investigating the Impact of Intervention Scenarios on Parasite Transmission Dynamics

We assessed the effectiveness of five management interventions that were shortlisted after discussions with interviewees and suggested by the veterinary official key informants (Table 3). Our assessment of effectiveness was based on the reduction in infection potential as defined by the amount of *L3*_*h*_ per kilogramme of herbage for the year 2018. Since carrying out health interventions for bharal would be logistically prohibitive, we concentrated on livestock-focussed interventions that might have an impact on parasite infections in both host populations. Interventions were housing and treating (with persistent anthelmintic). These are equivalent in our models in that they both simply interrupt egg supply to the pasture for a determined period. The intervention scenarios were divided into two types (i) pre-peak and (ii) early season. The peak infection day was identified by calculating the median date of the mode peak infection day from the GLOWORM-FL output in 1985–2018. Early season was defined both at a fixed time (related to predicted peak using the model, see Transmission Model—Predicting Pasture Infectivity Over Time) and much earlier than the pre-peak scenario (Table 3). The scenario with the greatest reduction in AUC*L3*_*h*_ relative to baseline (=no intervention) was selected as the most effective.

**Table 3:** Intervention scenarios to reduce infection and align livestock and wildlife health in the Kibber grazing system.

**Intervention**	Reasoning^*^	**Assumption**
*House or treat livestock 2 weeks prior to the peak infection.—Intervention 1*	Keeping livestock off pasture or treating them just before the peak might have a disproportionate impact on reducing the peak	There is a defined peak in infection that can be targeted
*House or treat livestock 4 weeks prior to the peak infection.—Intervention 2*	Same as above. However, the extended time period gives greater opportunity to reduce infection	Same as above
*House or treat livestock for 1 month (May) early in the season—Intervention 3*	Prevent the initial build-up of infection, which will disproportionately limit the increase in infection in the summer. May, driven by increasing temperature, is considered to be the beginning of the productive season in the Trans-Himalayas (61)	Given the harsh winters, we expect that warming spring and summer temperatures might fuel an increase in infectivity on pasture
*House or treat livestock for 1 month (June) early in the season—Intervention 4*	Same as above	Same as above. However, warmer temperatures in June than May are more suitable for larval development, and ceasing egg output then might have a larger effect
*House or treat livestock for 2 months (May–June) early in the season.—Intervention 5*	Same as above. However, the extended time period may be more effective at reducing infection	Same as interventions 3 and 4

*In each case, peak infection refers to the time of maximum L3_h_ availability, as predicted by the baseline (non-intervention) model.*

* *Reasoning column rationalises the reasons for the interventions, while the Assumptions column makes explicit certain conditions for this reasoning to be valid*.

## Results

### Livestock and Bharal Density and Distribution

We estimated a population size of at least 130 (130–164) bharal in the 27 km^2^ study area. Detection probability for observer 1 was 0.89 and for observer 2 was 0.73. This equated to a density of 4.81 (4.81–6.07) bharal km^−2^. In the same area, we recorded 545 sheep and 64 goats, representing a density of 22.6 small ruminant livestock km^−2^.

The FGDs revealed that livestock are herded to pasture each day and brought back to the village at night. They are stall-fed in pens during the harsh winters, and graze the pastures around the village through the rest of the year (including year-round when winters are mild). The Kibber livestock pasture (Figure 3) is the maximum total area grazed by Kibber's livestock throughout the year. These pastures are also where the area's only wild ungulate, bharal, graze. All FGD members unanimously agreed that bharal, even though they exhibit fission and fusion amongst groups, were present in the Kibber livestock pasture area throughout the year; indicating year-round pasture sharing. However, it is important to note that bharal habitat and groups exist far beyond the Kibber livestock pasture as well. Kibber's pastures were used for livestock grazing throughout the year in 2018 (as has been the case in recent years) with daily rotations to new grazing depending on where the stock had grazed the previous day. Herding duties are shared by members of livestock-owning households, with the rotation decided upon collectively by the villagers. The daily-grazed area is significantly smaller than the entire pasture area (around c. 5 km^2^/day). The eastern region of the Kibber livestock pasture is rarely grazed by livestock or bharal as it comprises high peaks.

### Management of Livestock Health

**Figure 4:**
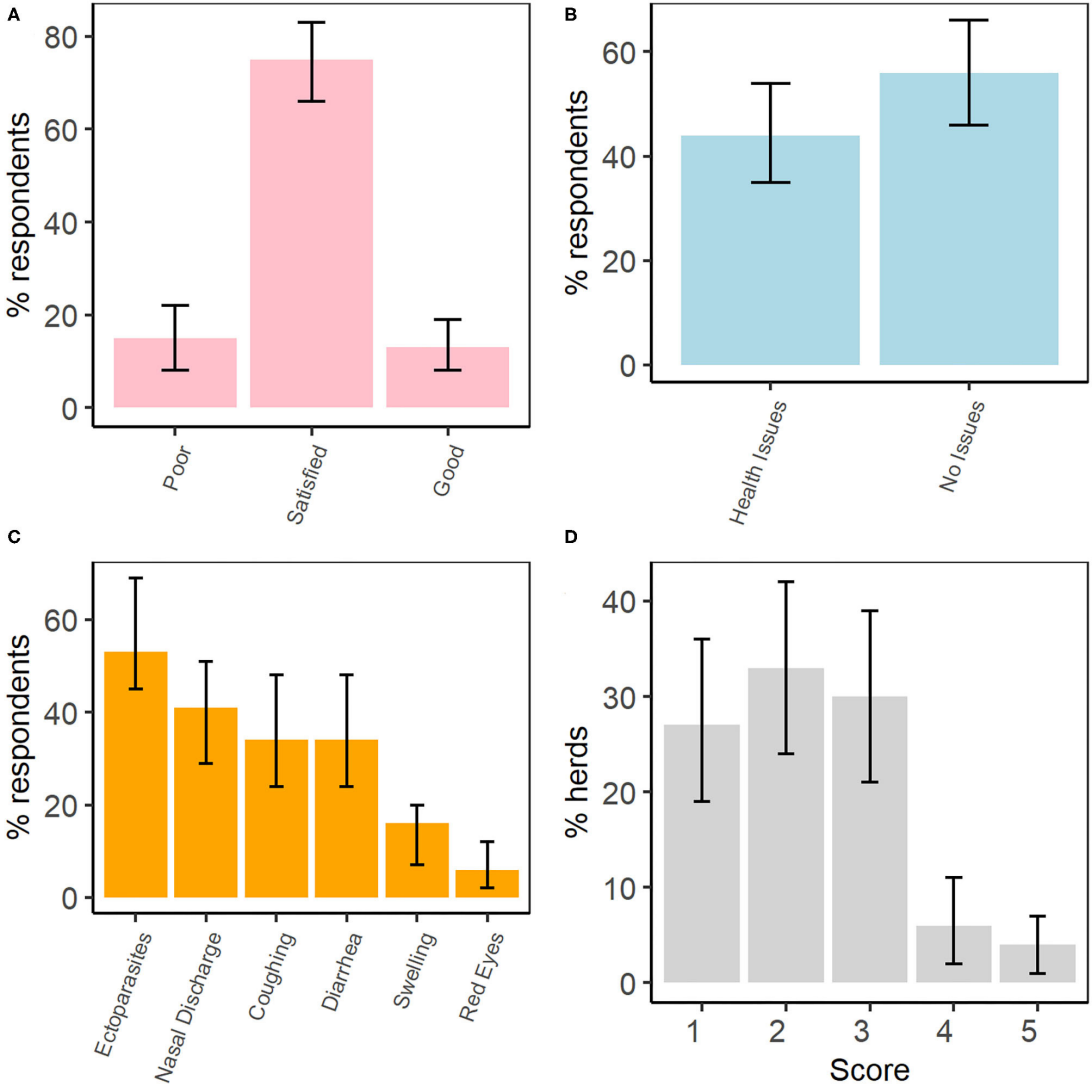
Selected results from the semi-structured interviews, including **(A)** livestock health perception, **(B)** reported presence of health problems in livestock, **(C)** list of health issues and symptoms in livestock, and **(D)** the averaged composite five-point check scores for livestock in each herd (*n* = 32 herds). Non-overlapping confidence intervals are interpreted as being statistically significant.

We interviewed 32 (57%) of 56 households with sheep and/or goats in Kibber. Most of the respondents (75%, 95% CI: 66–83%, *n* = 24) rated the health of their livestock to be satisfactory, with significantly fewer rating livestock health as poor or good (Figure 4A). Although over half of the respondents (56%, 95% CI: 46–66%, *n* = 18) claimed that their livestock had no disease or health issues, the rest (44%, 95% CI: 35–54%) identified issues, most commonly ectoparasites, nasal discharge, coughing and diarrhoea (Figure 4B). A few respondents gave examples of diseases of the eye, liver, and foot and mouth disease (FMD) as being prevalent. Of all the respondents, 20 believed that these diseases and symptoms are more prevalent in the winter, while the rest (*n* = 12) felt that prevalence is higher in the summer. When asked about the causes of these diseases, 10 interviewees did not know, while others attributed the onset of diseases to weakness (*n* = 7) or the cold weather (*n* = 6). Nearly all livestock herds (28/32) were reported to be vaccinated against FMD. According to the interviewees, no other vaccine was administered to their livestock.

All respondents were aware of ectoparasites and over half the respondents (*n* = 17) said their livestock had them. Of these, 11/17 respondents mentioned ticks, followed by lice (5/17). In fact, ectoparasites were listed most often by respondents as causing health issues in livestock (Figure 4C). The majority of respondents (*n* = 21) stated that ticks are responsible for weakness, and possibly death, in livestock. Moreover, nearly half the respondents (*n* = 15) claimed that ectoparasites are more common in winter, while 11 observed them to be present all year round, and six said they were more common in summer. No respondent had information about the occurrence of endoparasites and none of them treated their livestock against endoparasites. Nevertheless, Five-Point Check scores suggested negative health outcomes in livestock that were consistent with impacts of endoparasitism (Figure 4D). Lastly, both the veterinary key informants suggested that the annual pasture sharing with bharal is conducive to disease cross-transmission, particularly indicating the possibility of endoparasite transfer.

### Endoparasites in Bharal and Livestock

We analysed 30 unique sample days for livestock (126 pooled samples) and 38 unique sample days for bharal (115 pooled samples). Parasites identified in faecal samples included GINs (*Nematodirus* sp., *Trichuris* sp., *Strongyloides* sp., and other strongyle nematodes whose ova are morphologically indistinguishable from each other), trematodes (*Dicrocoelium* sp.), cestodes (*Moniezia* sp.), and protists (coccidial oocysts, *Eimeria* spp.) (Table 4). Some larvae were also observed, which were morphologically consistent with lungworm species, as well as ova resembling those of *Fasciola* spp., but because of inconsistent buoyancy of these species in saturated saline solution, levels of infection were not quantified. Endoparasite faecal density was significantly higher in livestock for *Emieria* spp., while strongyle nematodes were significantly higher in bharal (Table 4). Supplementary Material 2 shows the eggs per gramme (EPG) results for all endoparasites in livestock and bharal.

**Table 4:** Endoparasites in livestock and bharal (blue sheep).

		***Eimeria***	***Strongyloides***	**Strongyle GIN**	***Nematodirus***	***Trichuris***	***Moniezia***	***Dicrocoelium***
Blue Sheep (*n* = 115)	Prevalence (%)	32	10	52	2	-	25	–
	Range (EPG)	15–565	5–15	5-35	5	-	5–220	–
	Mean EPG (95% CI)	45 (41–49)	1 (0.9–1.1)	6 (5.6–6.2)	0.08 (0.06–0.1)	-	19 (17-21)	–
Livestock (*n* = 126)	Prevalence (%)	84	18	48	2	2	40	56
	Range (EPG)	5–15,600	5–15	5–35	5–15	10–20	5–215	5–65
	Mean EPG (95% CI)	635 (570–699)	1 (1.2–1.4)	3 (2.6–3.0)	1 (0.9–1.1)	0.2 (0.1–0.3)	15 (14.0–16.4)	0.8 (0.6–1.0)
Boot-strap *t*-test statistics		*t* = 3.59 df =126 *p* = 0.0004	*t* = 0.82 df = 237 *p* = 0.41	*t* = −3.39 df = 182 *p* = 0.0009	*t* = 3.99 df = 144 *p* = 0.0001	–	*t* = −0.669 df = 203 *p* = 0.51	–

*Livestock samples consisted of pooled counts from mixed herds comprising mainly sheep, with some goats, and could not be separated by species. Prevalence is consequently reported at the level of the pool and not the individual animal. EPG = eggs (or, for Eimeria spp., oocysts) per gramme; 95% CI: 95% bootstrapped confidence interval; GIN = Gastrointestinal nematode. Mean is the average egg density across positive pools*.

### Transmission Model

#### Predicting Pasture Infectivity Over Time

We used strongyle FEC as input to the GLOWORM-FL model (Table 4 and Supplementary Material 5). Strongyles were found in bharal and livestock throughout the year, albeit with variation in levels of egg output (Supplementary Material 5). Strongyle FEC were lower in the winter months for both hosts (days 0–50 and 300–365), peaked for bharal in late summer (around day 250), and remained fairly uniformly high for livestock through summer (days 180–275). Using information from the FGDs, we estimated that half of the strongyle eggs produced by livestock are deposited onto pastures, as they are housed for around 12 h per day.

**Figure 5:**
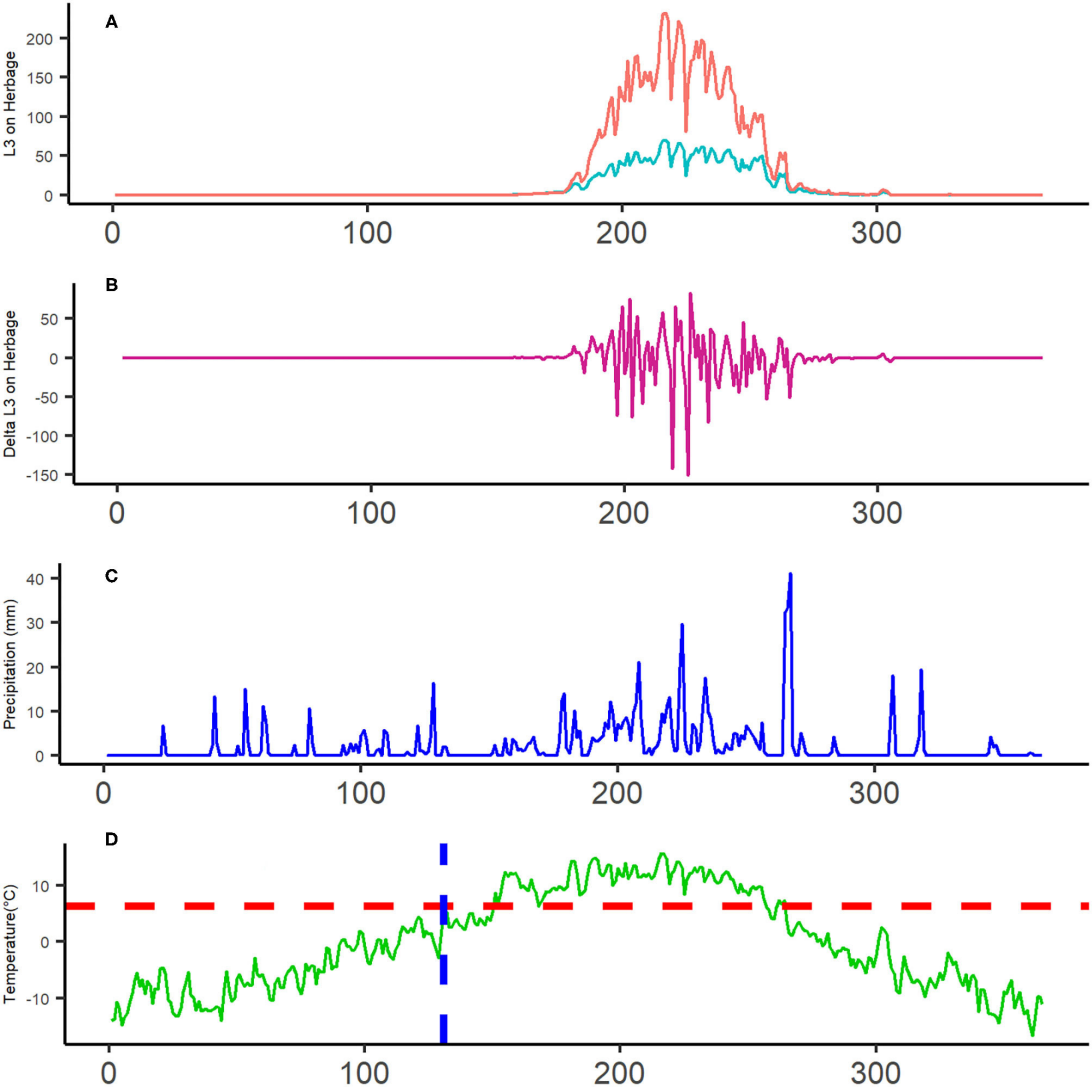
Panel graph for disease transmission dynamics in Kibber in 2018. The x-axis for each graph is day with 0 = 1st January 2018 and 365 = 31st December 2018; day 200 is late July. **(A)** GLOWORM-FL model output (number of *L3*_h_ per kilogramme of herbage) for individual hosts, Pink = livestock and blue = bharal. **(B)** Daily change in *L3*_h_ per kilogramme of herbage, using the data in **(A)**. **(C)** The amount of precipitation (mm) on a given day in 2018. **(D)** the average temperature (^0^C) on a given day in 2018. The vertical blue-dotted line on **(D)** indicates the first day on which larval development is possible, based on the lower development threshold of 6.6°C (horizontal red-dotted line).

The model predicted that infective *L3*_*h*_ larvae per kilogramme of herbage peak on pasture in summer, and that the contribution of livestock to infection potential on the pasture is higher than that of bharal (Figure 5A), in spite of lower average FEC (Table 4). Running the model for combined hosts (livestock and bharal) shows similar seasonality and summed magnitude. Infection was predicted to stay extremely low until towards day 180 of the year (i.e., July), reaching its peak just after day 200 and then tapering towards zero again by late September/early October (around day 270; Figure 5A). Daily change in *L3*_*h*_ per kilogramme of herbage abundance was variable through summer (Figure 5B) and not distinctly aligned with precipitation events, which occurred on most days (Figure 5C). *L3*_*h*_ levels per kilogramme of herbage began to increase around 40 days after the minimum threshold temperature for development was reached, coinciding with the steady increase in spring/summer temperature (Figure 5D).

#### Identifying Climatic Drivers of Predicted Infection Pressure

The area under the curve of *L3* over time (AUC*L3*_*h*_; Figure 5A sum of both the lines) was used as an index of overall pasture infectivity over the year. When a saturating amount of precipitation was applied, removing constraints of rainfall on larval availability, AUC*L*_3_ increased only marginally. Hence, the quotient $\frac{L_{3}AUC\;real\;precipitation}{L_{3}AUC\;high\;precipitation}\;$was 0.92. A quotient close to 1 indicates marginal influence of rainfall variability, and hence a more temperature-driven transmission system. This is consistent with Figures 5A,D, which shows that *L3*_*h*_ abundance per kilogramme of herbage rises gradually after the temperature exceeds the development threshold and peaks soon after peak annual temperature.

#### Quantifying the Contribution of Current Climate to Infection Pressure

**Figure 6:**
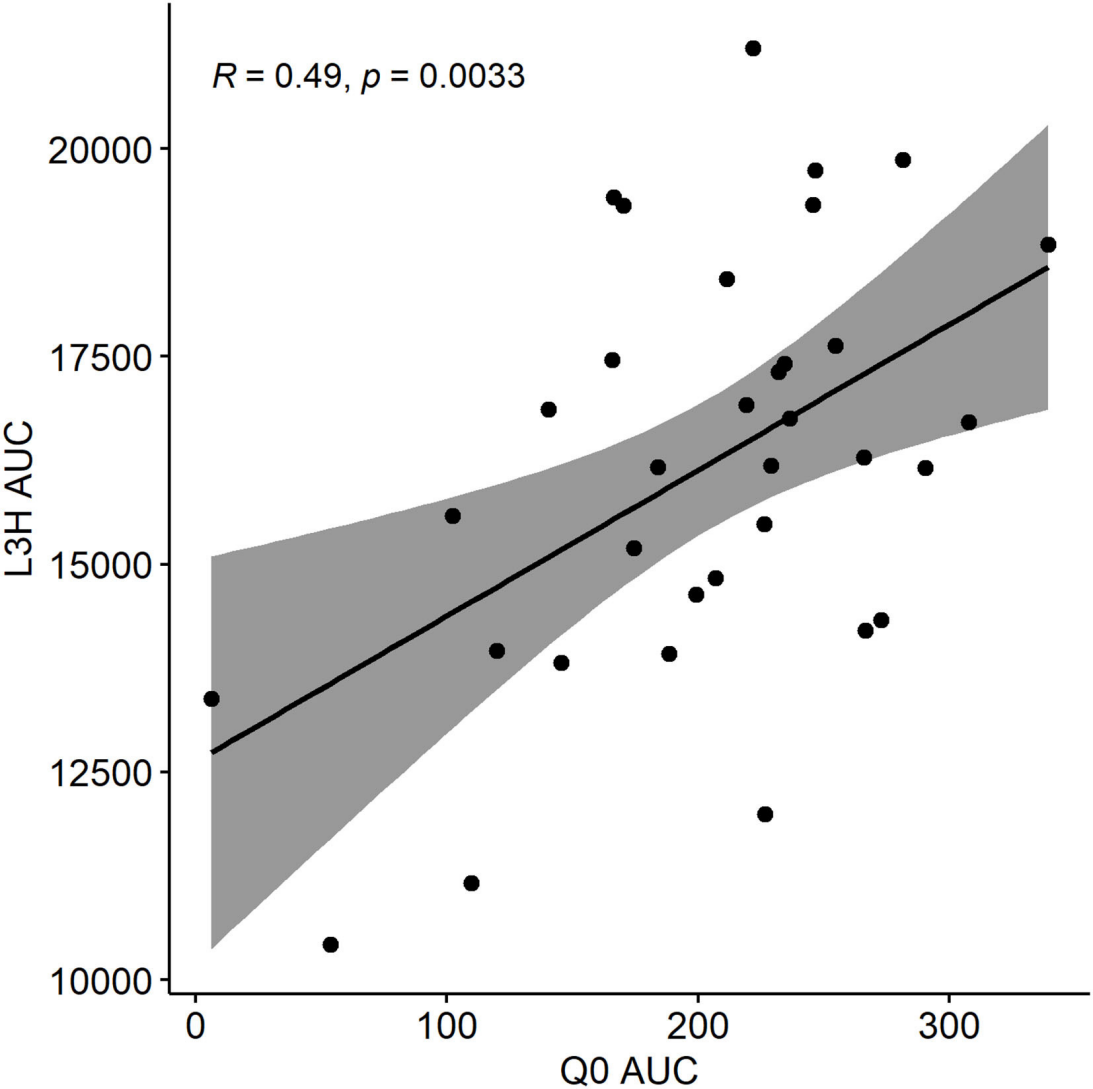
AUC*L3*_*h*_ area under the curve ~ AUC*Q*_0_area under the curve.

The time-explicit GLOWORM-FL model was used to predict total larval availability on pasture, taking into account variation in host faecal egg output and lags in development time of *L3*_*h*_, as well as weather. The *Q*_0_ model formulation, in contrast, isolates the climatic component of transmission only, with host and adult parasite factors held constant, and predicts how suitable each day is for parasite transmission success independently of historical pasture use and time lags between egg deposition and larval availability. In both cases AUC was used to aggregate outputs over the year. A perfect correlation between AUC*L3*_*h*_ in a given year and AUC*Q*_0_ in the same year would indicate that pasture infectivity was entirely explained by day-to-day variation in weather conditions. The correlation coefficient for AUC*L3*_*h*_ vs. AUC*Q*_0_ for the years 1985–2018 was 0.49 (Pearson's correlation test, *n* = 34, *p* = 0.003), suggesting that factors other than current weather alone (e.g., historical pasture use) are equally important in driving transmission potential (Figure 6).

To understand underlying trends and correlations in the parasite dynamics, we ran correlations between climatic variables and model outputs across time (Table 5). The $\frac{L_{3}AUC\;real\;precipitation}{L_{3}AUC\;high\;precipitation}$ quotient showed a tendency to increase over the years (Table 5). This indicates that over this period precipitation became less important as a constraint to transmission, and that temperature is increasingly the main driver of *L3*_*h*_ availability on pasture. Additionally, AUC*Q*_0_ increases with time but not AUC*L3*_*h*__−_. AUC*L3*_*h*__−_ per kilogramme of herbage (infection) and AUC*Q*_0_ for 1985–2018 are given in Supplementary Material 6.

**Table 5:** Correlation matrix between climate data set and model outputs for the years 1985–2018.

	**Time**	**Model Outputs**			**Climate data set**	
	***Year***	***AUCL_**3**_***	***Quotient***	***AUCQ_**0**_***	***Mean Temp***	***Mean Precip***
*Year*	1					
*AUCL_3_*	r = 0.083; *p* = 0.64	1				
*Quotient*	r = 0.75; *p* < 0.001	r = 0.45; *p* = 0.007	1			
*AUCQ_0_*	r = 0.67; *p* < 0.001	r = 0.49; *p* = 0.003	r = 0.83; *p* < 0.001	1		
*Mean Temp*	r = −0.44; *p* < 0.001	r = 0.31; *p* = 0.078	r = −0.41; *p* = 0.015	r = −0.25; *p* = 0.16	1	
*Mean Precip*	r = 0.74; *p* < 0.001	r = −0.019; *p* = 0.92	r = 0.77; *p* < 0.001	r = 0.66; *p* = <0.001	r = −0.52; *p* = 0.001	1

*AUCL3_h_ = area under the curve of predicted infective larval (L3_h_) density on herbage, a measure of overall nematode infection pressure. AUCQ_0_ = area under the curve of Q_0_, quantifies the contribution of current weather to infection pressure. Quotient = $\frac{L_{3}AUC\;real\;precipitation}{L_{3}AUC\;high\;precipitation}.$Temp = Temperature. Precip= Precipitation. Correlations in grey are non-significant, green are significant positive correlations, and blue are significant negative correlations (α = 0.05)*.

**Figure 7:**
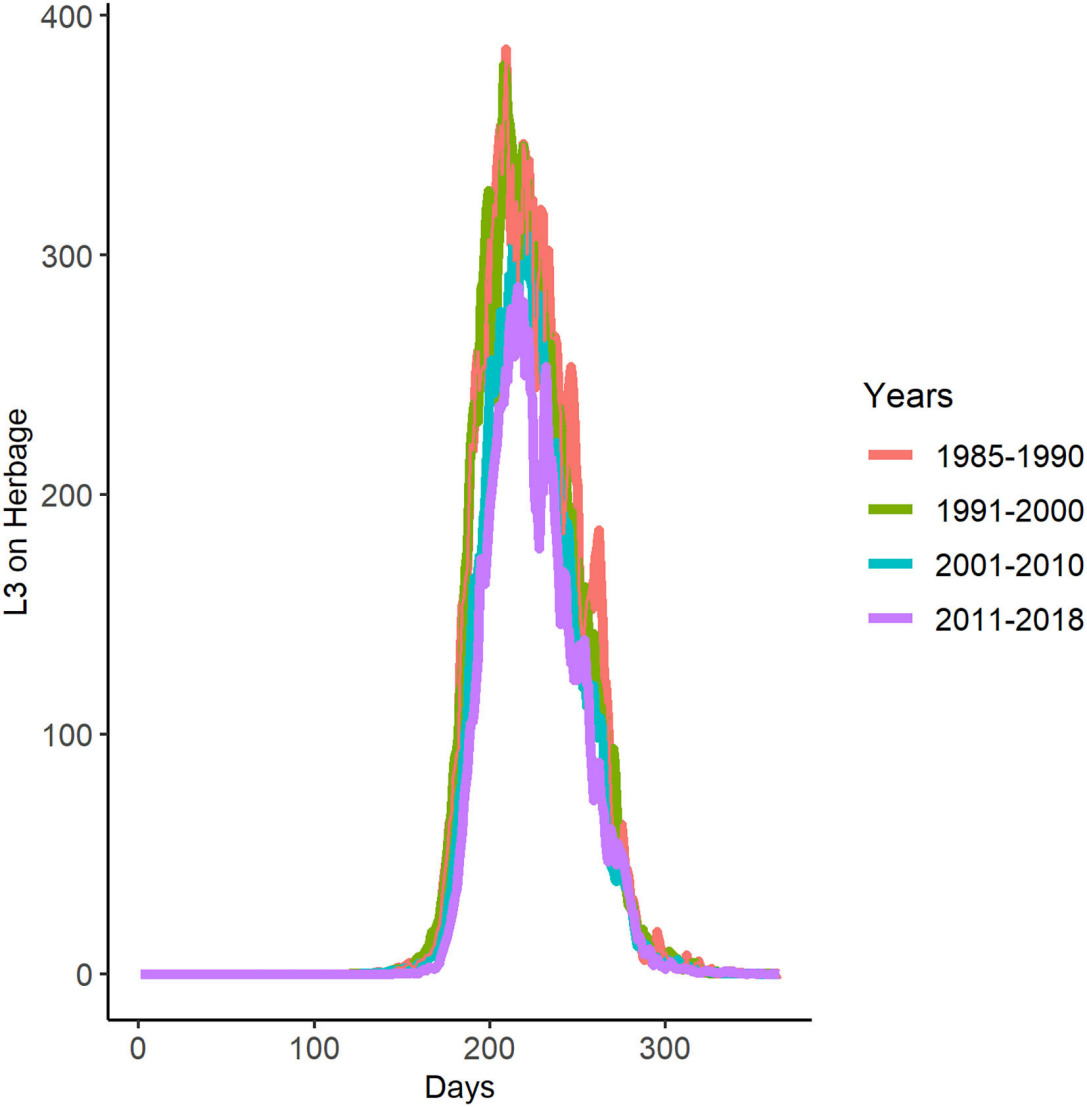
Decadal averaged GLOWORM-FL output for the years 1985–2018. The x-axis represents the days of each year starting with 0 = 1st January and 365 = 31st December.

Applying GLOWORM-FL to weather data for each year between 1985 and 2018 showed little change in the seasonal pattern of *L3*_*h*_ infective larvae per kilogramme of herbage on pasture over time. The general pattern conformed to the 2018 output (Figure 5A); with a single peak in late summer (Figure 7 and Supplementary Material 7). In the period 1985–2010, the mean peak infection day was 216 (median 215 and range 201–234). Therefore, for all five interventions (see below), the dates were defined with respect to an infection peak on day 216. Consequently, early season suppression months were selected as May and June, as that represents the transition of spring into summer, which also sees the initial rise in *L3* (Figures 5A,D dotted lines).

#### Investigating the Impact of Intervention Scenarios on Infectivity Dynamics

**Figure 8:**
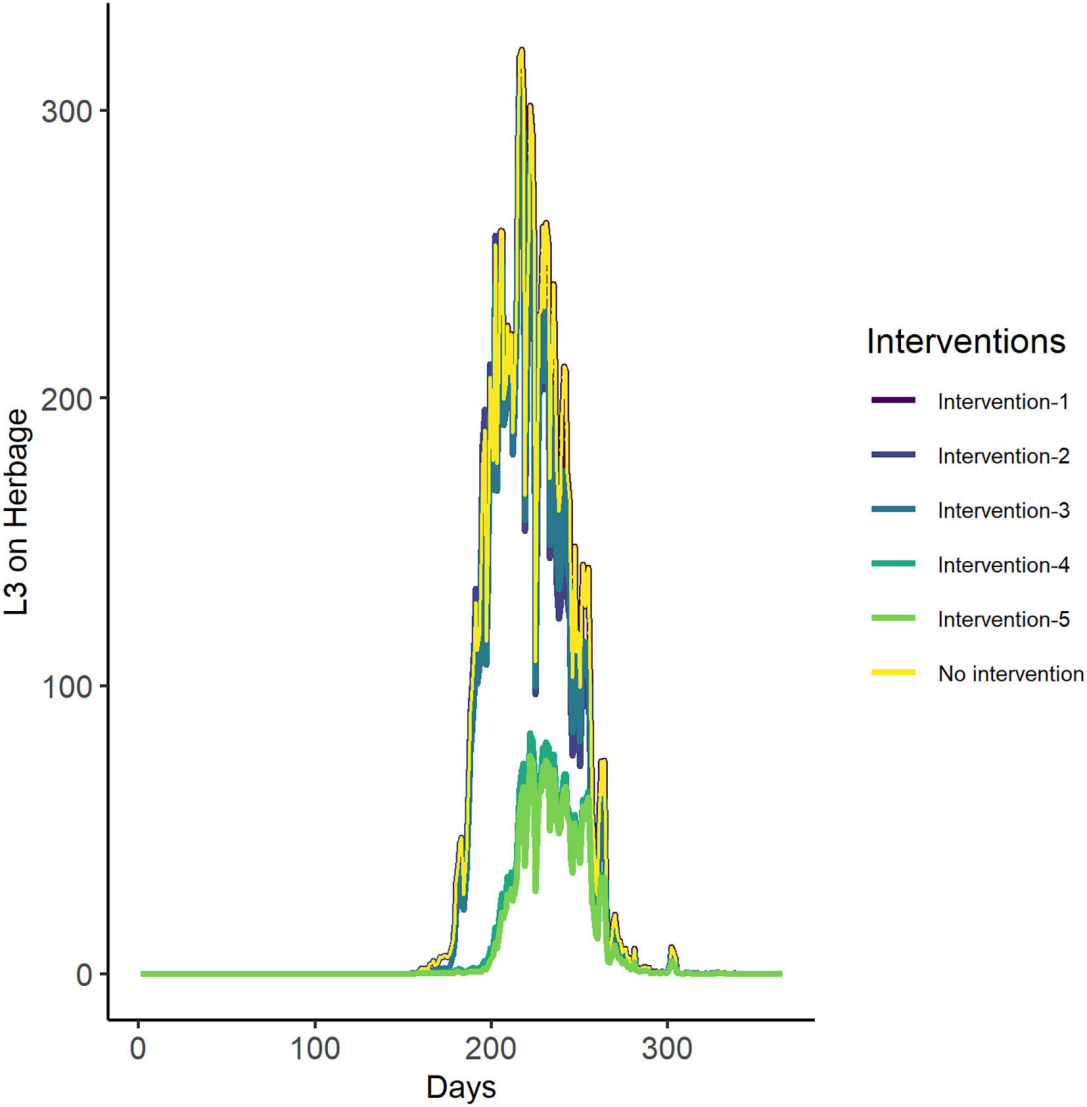
The GLOWORM-FL output for all interventions (as stated in Tables 3, 6) and no intervention, for the year 2018.

The first three intervention scenarios had little predicted impact on overall pasture infectivity. Interventions 4 (house or treat for 1 month—June- early in the season) and 5 (house or treat for 2 months—May and June—early in the season), however, significantly lowered the predicted number of infective larvae on pasture. The most successful interventions were therefore to house or treat livestock for 1 or 2 months (June, or May and June) early in the season (Figure 8, Table 6).

**Table 6:** Intervention scenarios and their impacts in reducing infection.

**Intervention**	**AUC Intervention**	**% reduction of AUC**
*No Intervention*	14,201	–
*House or treat livestock 2 weeks prior to the peak infection—Intervention 1*	13,094	7.80
*House or treat livestock 4 weeks prior to the peak infection—Intervention 2*	12,759	10.15
*House or treat livestock for 1 month (May) early in the season—Intervention 3*	12,668	10.80
*House or treat livestock for 1 month (June) early in the season—Intervention 4*	3,295	76.79
*House or treat livestock for 2 months early in the season—Intervention 5*	2,978	79.03

## Discussion

We investigated disease transmission risk through a socio-ecological lens in an Indian trans-Himalayan multi-use landscape. We had the dual aim of understanding the characteristics of the system with respect to parasite transmission and, based upon that, evaluating the effectiveness of potential climatically-adapted interventions to align livestock and wild ungulate health.

### Host Distribution, Social Factors to Manage Livestock Health and Endoparasites

By engaging with herders, we found that livestock and bharal share the pastures around Kibber throughout the year, enabling indirect contact via pasture sharing. Host overlap is a significant factor in the epidemiology of several shared pathogens (62), particularly GINs (63). Crucially though, the scale and the nature of overlap determine whether and how transmission will actually occur (11). Finer scale data on livestock and bharal distribution and movement on a daily or seasonal basis, would enable more sophisticated spatial modelling of disease transmission potential and also a wider range of management options such as pasture rotations. Nevertheless, given reported continual pasture use by both bharal and small ruminants throughout the year, it seems reasonable to make a starting assumption of complete overlap between populations.

Endoparasites were not raised as a common issue during group discussions or interviews, despite FEC data and Five-Point Checks suggesting their presence and potential negative impacts. Consequently, no interventions were in place to manage infection and transmission. Because endoparasites are not visible, they are often underestimated by herders, relative to the more obvious ectoparasites and in spite of health indicators suggestive of infection. This seems to be the case here. Of concern is that livestock in this region have been shown to be overstocked, with compromised productivity (29), which would accentuate the negative impacts of even moderate parasite burdens. There is a need to more fully assess the diversity and infection intensity of endoparasites in both domestic and wild ungulates in the region, and their impacts on health, production and fitness. Where impacts are likely, it will be important to raise awareness of the issue in order for proactive management strategies to be adopted where necessary (64).

In the present limited study, strongyle GINs density in pooled faecal samples was relatively low (5–15 EPG, whereby the threshold for veterinary intervention in more intensive livestock-rearing systems might be 200–300 EPG), which may be testament to the general management of goats and sheep in Kibber, including housing for around 12 h at night-time (thus potentially reducing pasture contamination by 50%), relatively low overall stocking densities of around 23 head per km^2^ (equivalent to 0.23 per hectare, around 30-50x lower than typical stocking densities in western Europe, for example), frequent daily movement to fresh grazing within the overall grazing area, and a relatively short parasite transmission season.

Low average egg density can conceal significant effects of parasitism in some individuals due to parasite overdispersion (39, 65), however, especially when hosts are on a low plane of nutrition (66). We did find evidence of poor health in sheep and goats in Kibber using the Five-point check, which is calibrated to detect the physiologically detrimental consequences of GINs, while not being specific to them. This could be further reducing productivity of the overstocked livestock in Kibber (29). Veterinary officials identified shared livestock-bharal grazing as conducive to disease transmission, specifically GINs, and FECs confirmed a range of endoparasite taxa were present in both hosts. This is a concern because exploitative competition between bharal and resident livestock has been shown to reduce survival of young bharal individuals in Kibber (34, 35). In wild ruminants, GINs have been shown to correlate with poor body condition even at low levels (67), and to reduce fecundity (68). Therefore, it is plausible that GINs might impact negatively on bharal fitness, especially if livestock act as alternative hosts and supply infection even at low bharal population density.

This potential for disease spill-over from livestock to bharal could have implications for the conservation of bharal and control of livestock diseases. For instance, a livestock-transmitted virus, Peste des Petits Ruminants Virus (PPRV), killed a significant proportion of the Critically Endangered Mongolian Saigas (*Saiga tatarica mongolica*) in 2016–17 (69). Spill-over into wildlife can further undermine outbreak control spilling back into livestock. Although GINs do not generally cause disease outbreaks with high levels of mortality, impacts on host health and fitness can be considerable (see above), while also potentially exacerbating impacts of other pathogens through increased susceptibility (70). However, there is a need to better investigate the physiological effects of GINs within hosts in Kibber before deciding whether interventions are required, and of what kind.

### Predicting Pasture Infectivity Over Time and Identifying Climatic Drivers of Predicted Infection Pressure

Using the GLOWORM-FL model, we predicted that although per-capita livestock contributions to pasture loads were lower because they were housed for 12 h a day, livestock made a greater contribution to pasture infectivity than bharal. This is likely to be due to the higher density of livestock per km^2^. Given year-round pasture-sharing, this suggests the potential magnitude of parasite transmission from livestock to bharal is significantly higher than *vice versa*, whilst not precluding some transmission from bharal to livestock. Model simulations predicted that pasture infectivity consistently peaks in late summer, which suggests maximal transmission of GINs at this point and that mature infections are carried over into winter. This has the potential to compromise host health in the harsh winter months; a time of year when ungulate body condition is often poor (36). In Svalbard reindeer, for example, GIN infection was associated with poor body condition in winter and subsequent low fecundity (68). However, contribution of GINs to poor body condition in bharal and hardy livestock breeds in the trans-Himalayan region is unclear, especially given the low FECs observed here.

Additionally, the model suggested that the seasonal increase in the number of infective larvae on herbage (*L3*_*h*_) occurs after a period of steady increase in temperature. Infective larvae on pasture first appear on day 131, however peak infection is not achieved until around day 216 (around 3 months later). This “slow burn” seasonality in parasite transmission could be driven by the modest fecundity of *Teladorsagia*, which is the GINs modelled in our study (cf. the more fecund *Haemonchus*), moderate temperatures (hence slow development), and low evapotranspiration (therefore a more prolonged impact of precipitation events on transmission).

In the absence of information on GINs species composition, the transmission model was calibrated to *Te. circumcincta* as this species is well-studied and has a climate envelope appropriate to the region and broadly similar to other species also common in small ruminants in temperate and montane environments, such as *Trichostrongylus* spp (47). However, predictions may have been different for other species. For instance, *Marshallagia* spp., although not found in our samples, is common in Chamois *Rupicapra rupicapra* and Alpine ibex *Capra ibex* across the European Alps, mainly during the winter (71), and differs in response to temperature (72). Therefore, further studies taking advantage of molecular approaches such as nemabiome (73) would be beneficial to identify specific parasites and their host distribution, enabling species-specific predictions to be made. These would, however, be subject to additional parameter uncertainty due to lack of data on response norms for many nematode species, especially in wildlife, and alternative approaches are needed [e.g., (74)]. Additionally, hypobiosis can affect the phenology of gastro-intestinal nematodes including in wild ungulates (75), and should be considered if found to occur in this system.

### Quantifying the Contribution of Current Climate to Infection Pressure and Investigating the Impact of Intervention Scenarios on the Disease Transmission Dynamics

By comparing GLOWORM-FL with *Q*_0_ model outputs for the years 1985–2018, we found that in Kibber the historic worm burden and the developmental delay between egg and *L3* on herbage were as important as current weather in driving *L3*_*h*_ levels (Figure 6). This suggests that current climate data alone are of limited use to predict infection pressure. Infection seasonality remained similar across 1985–2018, with a distinct late summer peak in pasture infectivity at a very similar time between years. The fact that over this period, AUC*Q*_0_ seems to increase but not AUC*L3*_*h*_, is also consistent with a “slow burn” system; there might be more days with conducive weather for worm development, but they are not strung together in a way that translates to higher *L3* abundance (Table 5). We also found an increasing trend in the quotient $\frac{L_{3}AUC\;real\;precipitation}{L_{3}AUC\;high\;precipitation}\;$between 1985 and 2018 (Table 5), which indicates that rainfall is increasingly not a limiting factor for GINs development and transmission, and that temperature is the primary (climatological) limitation for transmission in this area.

The fact that temperature is a better predictor of *L3*_*h*_ abundance in this region than rainfall opens up the potential to use temperature to inform risk assessment. For example, stakeholders can use rising summer temperatures (above the minimum development threshold of 6.6°C; Figure 5D) as an approximate predictor for increasing infection pressure. The approach taken here, to assess the relative importance of temperature vs. rainfall in predicting transmission potential based on *L3*_*h*_ abundance, could also be used elsewhere to produce pragmatic models for risk assessments in livestock systems.

As the aim of our modelled interventions was to reduce the total infection pressure and infection peaks in the study period were reached fairly consistently in the day range 201 to 234 (mid-July to mid-August), the date appears to be a useful proxy for temperature as a cue for interventions (Tables 3, 6). The finding that interventions four and five (treat livestock or keep them off the pasture for 1 month in June, or 2 months in May and June, thus early in the season) were the only scenarios achieving a discernible impact on infection magnitude is entirely consistent with a “slow burn” system. Historical pasture use (i.e., egg shedding) over weeks or months, along with accumulated periods of larval development, govern the standing crop of infective larvae on pasture in these types of system. This is in contrast to systems in which specific climatic events (e.g., rainfall on *Haemonchus* eggs when temperature is well above threshold) or seasonal host movements [e.g., (76); absent here] create discrete critical time points at which interventions might be focused (77).

This result is similar to many temperate systems, where the most effective intervention is to treat early in the season, i.e., “early season suppression,” on the basis that it is these eggs that ignite the system and by preventing them being shed, pastures are kept clean for longer and infective larvae lack the chance to reach a high peak (78). Intervention 3 (early-season suppression in May) had discernibly lower impact than intervention 4 (early-season suppression in June) because in the first month after the development threshold is reached (late April into May), temperature is still low, such that stopping egg inputs then made little difference to eventual pasture contamination. A similar outcome could therefore be achieved with a shorter intervention, if this is well-timed in relation to parasite development potential, and the model can enable intervention times and weather cues to be optimised to the system in hand.

Cessation of egg output could be achieved by anthelmintic treatment or by housing. Housing livestock for 1 or 2 months early in the season might be unrealistic for various logistical and socio-cultural reasons. These include: (i) a shortage of fodder in the region and reliance on continual grazing of pastures; (ii) due to the short vegetative growth season, the quality and variety of vegetation is at its peak during summer, which herders try to exploit before the onset of the winter; (iii) due to their Buddhist faith, the herders value the right to life of every sentient being and hence could feel that restricting livestock movement out of choice, for extended periods of time, would impinge on these rights. Treating all animals for over two months, on the other hand, would be expensive and could favour the development of anthelmintic resistance (79). Nonetheless, we evaluated this scenario to confirm the “slow-burn” nature of the system. Importantly, a key research need is to investigate if the magnitude, direction and level of infection cross-transmission is high enough to be physiologically detrimental for the hosts (80). If not, then interventions may not be required.

### Lessons for Kibber: Integrating Inputs From Interviews With Model Outputs

Temperature can be used by herders in Kibber as a cue to be vigilant and mitigate against effects of GINs in their herds (Figures 5, 7 and Table 6). Nonetheless, rather than taking an effective, yet arguably impractical preventive measure, it might be more cost-effective to use timely and reactive methods such as selective treatment of individuals showing signs of parasitism. This may achieve disproportionate health improvements and epidemiological benefits using lower levels of anthelmintic treatment (81). Further adaptations to our model could enable simulation of the likely impact of selective treatments (82). Livestock-holders and veterinary officials could use the Five-Point Check to check for parasitism in sheep and goats and identify vulnerable individuals (37). Combining the understanding of transmission seasonality and magnitude from GLOWORM-FL with identification of those livestock individuals showing signs of parasitism, could help in selecting times and individuals at which to target treatment. This would reduce the onset of anthelmintic resistance and is a cost-effective way to treat herds where drug supplies are limited, such as in remote areas like Kibber. This has the potential to reduce the onward transmission of GINs from livestock to bharal while improving livestock health.

Additionally, our coarse analysis would need to be followed up by a finer-scale (ideally individual-based) analysis of disease and GIN loads both for livestock and wildlife, so that the heterogeneity within herds and its spatio-temporal variation can be assessed. This would enable assessment of the potential benefits of a targeted treatment approach, both for livestock and bharal. However, such interventions need consensus about their effectiveness in order to trigger channelling of resources, which often takes time to build evidence, engagement and momentum. Studies such as this one provide both the scientific foundation and the foundation of trust between researchers and herders, which could enable more targeted and effective disease control strategies in future.

## Conclusion

Using a robust strategy rooted in understanding of system dynamics under a changing climate, we find that the Trans-Himalayan Kibber pastures can be characterised as a “slow-burn” parasite system that is temperature-driven. Over the years 1985–2018, this feature appears to be increasingly reinforced, while the seasonality of parasite transmission is relatively constant and predictable. Consequently, early-season suppression of GINs egg output form livestock is the most effective strategy to limit infection pressure both for livestock and bharal, particularly if consistently applied from year to year, for long enough to make a difference to overall larval abundance.

Finally, looking beyond Kibber, our study provides a transferable multi-pronged approach to investigating disease transmission risk through a socio-ecological lens in a multi-use landscape. By highlighting that disease is a socio-ecological concern, we emphasise that its understanding and management is best considered from an interdisciplinary perspective. Our holistic approach combines ecological and social knowledge to understand parasite transmission in a multi-use landscape and provide a scientific basis for interventions. Not only can this protect herders' livelihoods but also conserve wild ungulates.

## Data Availability Statement

The original contributions presented in the study are included in the article/Supplementary Material, further inquiries can be directed to the corresponding author/s.

## Ethics Statement

The studies involving human participants were reviewed and approved by University of Bristol. Written informed consent for participation was not required for this study in accordance with the national legislation and the institutional requirements. The animal study was reviewed and approved by University of Bristol. Written informed consent for participation was not obtained from the owners because we received oral consent as our respondents were not literate. We have elaborated this in our manuscript.

## Author Contributions

MK, EM-G, KS, and EM conceived the idea of the project. MK, AK, and RR conducted the field work. ED contributed significantly to the analysis. HR provided initial R code for modelling and developed additional parameters for the *Te. circumcincta* Q0 model. MK and EM-G wrote the first draft of the manuscript. All authors commented on and refined subsequent drafts.

## Conflict of Interest

The authors declare that the research was conducted in the absence of any commercial or financial relationships that could be construed as a potential conflict of interest.

## Publisher's Note

All claims expressed in this article are solely those of the authors and do not necessarily represent those of their affiliated organizations, or those of the publisher, the editors and the reviewers. Any product that may be evaluated in this article, or claim that may be made by its manufacturer, is not guaranteed or endorsed by the publisher.
