# Supplementary material for: Predicting Parasite Dynamics in Mixed-Use Trans-Himalayan Pastures to Underpin Management of Cross-Transmission Between Livestock and Bharal
Source: Front Vet Sci. 2021 Sep 29;8:714241. doi: 10.3389/fvets.2021.714241 (PMC8511524; doi:10.3389/fvets.2021.714241)
Supplement: Supplementary file 1 [file Data_Sheet_1.PDF]

Supplementary Material 1- Questionnaire

Preamble

Before starting the interviews, each respondents was told that the survey's aim was to understand livestock health and management practices in Kibber.

Each respondent was told that their identity will be kept anonymous and will not be identified by name in any publications and communication. They were also told that this study is part of Munib Khanyari's PhD thesis that aims to understand how species movement along-with environmental factors (like weather) affect disease transmission between wild and domestic ungulates. They were also told that the results will be published in internal reports, publicly available peer-reviewed, scientific journals. Publications will solely refer to the village/area where the survey was conducted, not individuals, unless requested not to do so by the respondents. After all this, we asked each respondent for permission to conduct the interview. Consent was orally recorded. We also explained that they can withdraw at any time. We referred to shoats when saying livestock.

Semi-structured interview questions:

Q1. How do you rate your livestock's health? Satisfactory, good, bad, other?

Q2. Do your livestock have health issues?

Q3. What kind of health issues do your livestock have? What causes them?

Q4. What kind of treatment/management do you employ against health issues?

Q5. Do your livestock have ectoparasites? When do they occur? What is their impact?

Q6. Do your livestock have endoparasites? When do they occur? What is their impact?

Q7. Across the year, where are bharal? Can you help us make a map of this?

Q8. How are your livestock distributed across the year around Kibber? Can you help us make a map of this?

After these directed questions, interviewees were allowed to speak freely on any aspects around livestock health and management practices.

Supplementary Material 2 – FEC raw data (attached excel)

Supplementary Material 3 – Ungulate Analysis

To estimate the number of groups ($\hat{G}$) of blue sheep, we fit the 'mt' model using the function BBRecap with a 'uniform prior' for each species. We used the 'mt' model because detection probability was expected to be different across the two surveys (Suryawanshi et al. 2012). We used uninformed uniform priors because this is the first effort to use this method in this landscape for blue sheep. We did 10,000 mcmc iterations with 1000 burn-in. Further details on model fitting are available in Fegatelli and Tardella (2013).

The estimated detection probability by model 'mt' for occasion one and two was interpreted as the detection probability for observer teams one and two. We estimated the total population of each ungulate species (N_{est}), as a product of the estimated number of groups ($\hat{G}$) and the estimated mean group size (μ). To estimate the confidence intervals of their population using the variance in estimated number of groups and the mean group size, we generated a distribution of estimated group size by bootstrapping it 10,000 times with replacement. A distribution of estimated population for each ungulate species(N_{est}) was generated by multiplying 10,000 random draws of estimated number of groups ($\hat{G}$) weighted by the posterior probability and draws of mean group size (μ). The median of the resultant distribution was the estimated ungulate population (N_{est}) and the 2.5 and 97.5 percentiles were used as the confidence intervals. The survey area was calculated by delineated the entire survey area of Google Earth Pro.

Supplementary Material 4 – Model Parameters

Table 1. Model parameters for *Teladorsagia Circumcinta* for the GLOWORM-FL Model

Parameter	Estimate*	Data Source**
$\boldsymbol{\delta}$	−0.02085 +0.00467T (F1,10 = 76.57, p < 0.001, R2 = 0.88, R2 adj = 0.87)	Rose et al. (2015)
$\boldsymbol{\mu_1}$	exp(−1.62026−0.17771T + 0.00629T2) (F2,2 = 6.27, p = 0.27, R2 = 0.93, R2adj = 0.78)	
$\boldsymbol{\mu_2}$	Same as above	
$\boldsymbol{\mu_3}$	10 x μ_4	
$\boldsymbol{\mu_4}$	exp(−4.58817−0.13996T + 0.00461T2) (F2,12 = 43.55, p < 0.001, R2 = 0.88, R2adj = 0.86)	
$\boldsymbol{\mu_5}$	Same as μ_3	
$\boldsymbol{m_1}$	0.21, P≥ 2<br><br>0, P < 2 and $\sum_{i=-7}^{t}\frac{P_i}{E_i} < 1$<br><br>0.025, P < 2 and $\sum_{i=-7}^{t}\frac{P_i}{E_i} \geq 1$	
$\boldsymbol{m_2}$	exp(−5.48240 +0.45392T−0.01252T2) (F2,1 = 442.9, p = 0.034, R2 > 0.99, R2 adj > 0.99)	
**C**	0.1, $\sum_{i=7}^{t}\frac{P_i}{E_i} < 1$<br><br>0, $\sum_{i=7}^{t}\frac{P_i}{E_i} \geq 1$	

*T, temperature(◦C); P, total daily precipitation(mm); E , total daily evapotranspiration (mm).
** Rose et al. (2015) obtained these parameters from several published studies. The exact list can be seen in Rose et al. (2015).

Supplementary Material 5 – EPG for Blue sheep and Livestock

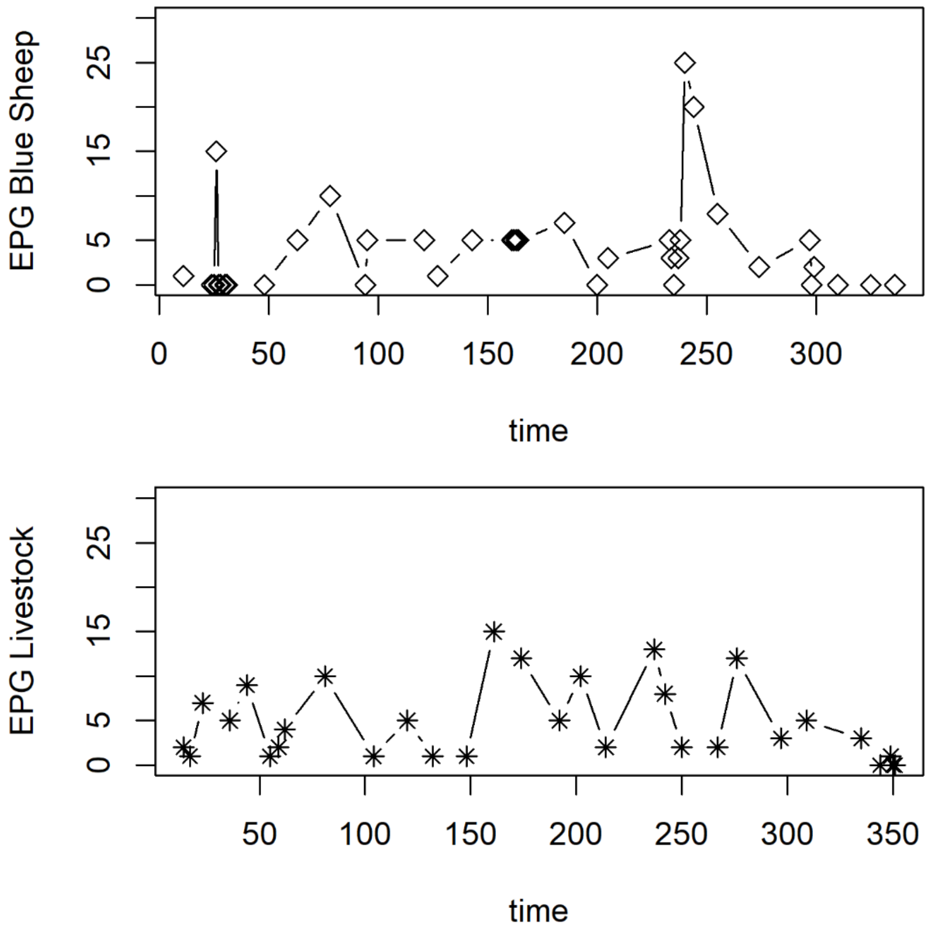

Figure 1. Line graphs showing the FEC (faecal egg count) EPG (eggs per gram) of strongyle sp. in Blue sheep and Livestock. These were used as inputs to the GLOWORM-FL model. The X-axis refers to days with 0 = 1st January 2018 and 365 = 31st December 2018.

Supplementary Material 6 – L3AUC and Q0AUC outputs for 1985-2018 (attached excel)

Supplementary Material 7 – GLOWORM-FL output (L3 infective larvae on herbage) for the years 1985-2018.
