# Supplementary figures and images for: Predicting Parasite Dynamics in Mixed-Use Trans-Himalayan Pastures to Underpin Management of Cross-Transmission Between Livestock and Bharal
Source: Front Vet Sci. 2021 Sep 29;8:714241. doi: 10.3389/fvets.2021.714241 (PMC8511524; doi:10.3389/fvets.2021.714241)

**Glowworm output 2018-1985 Kibber**

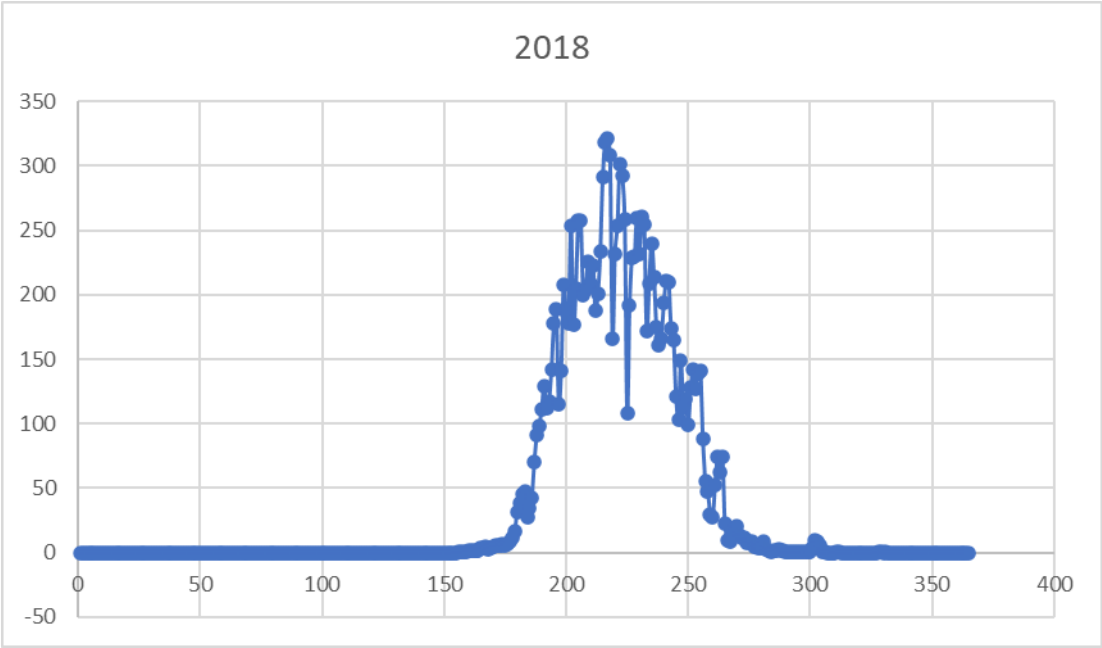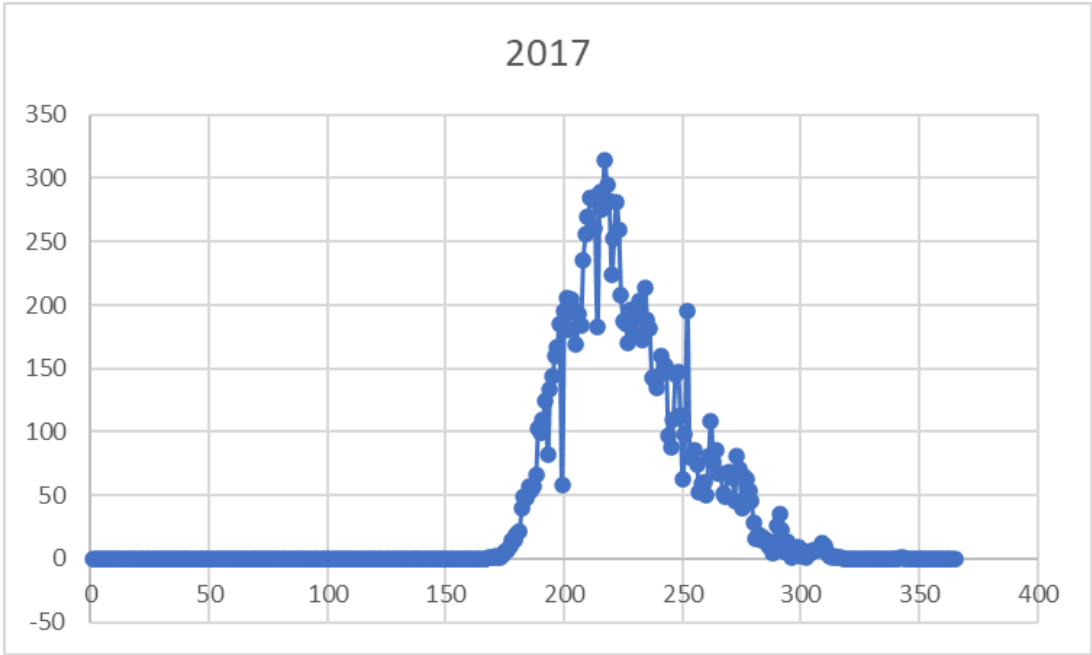

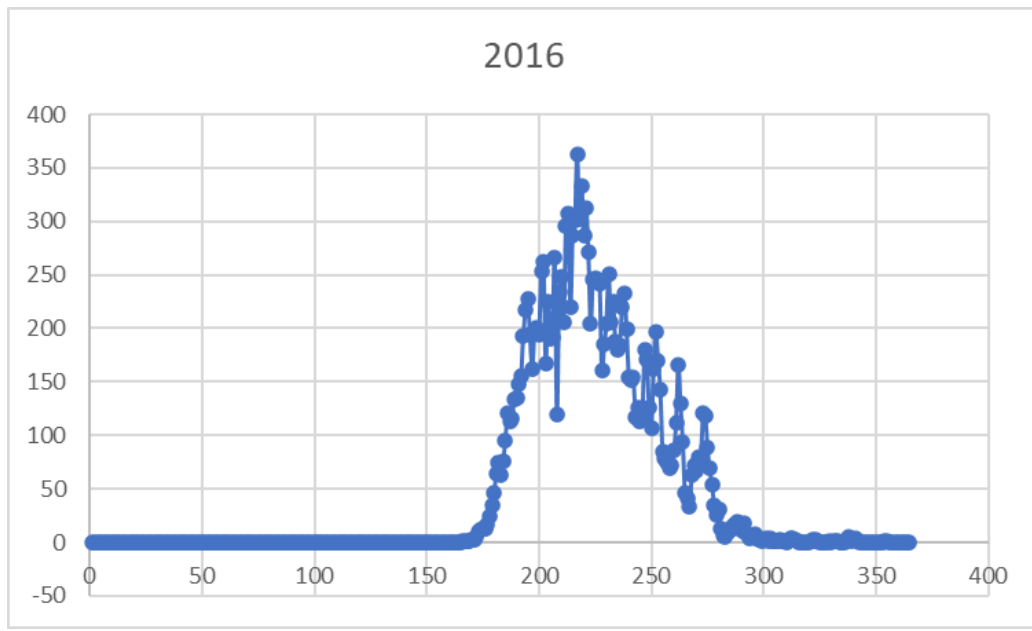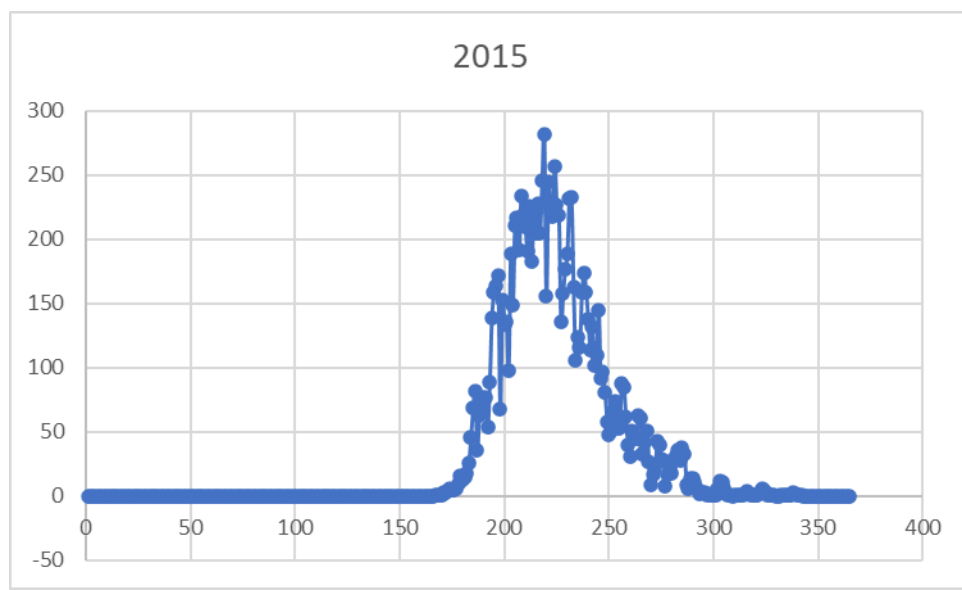

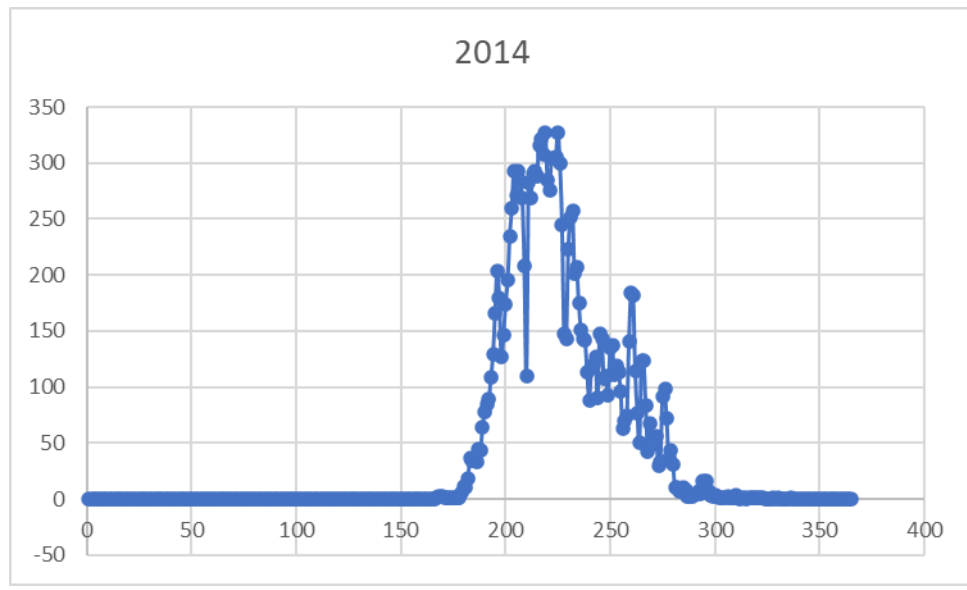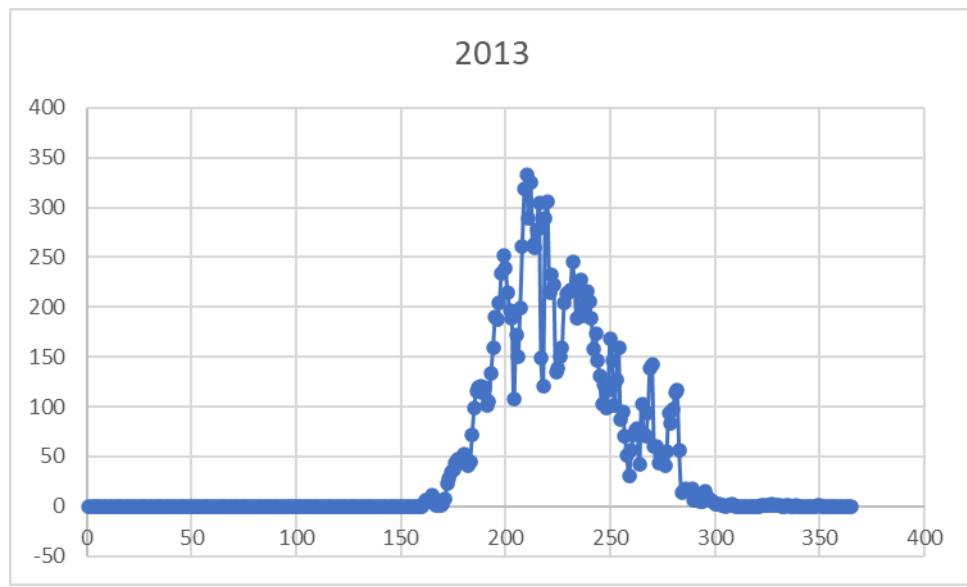

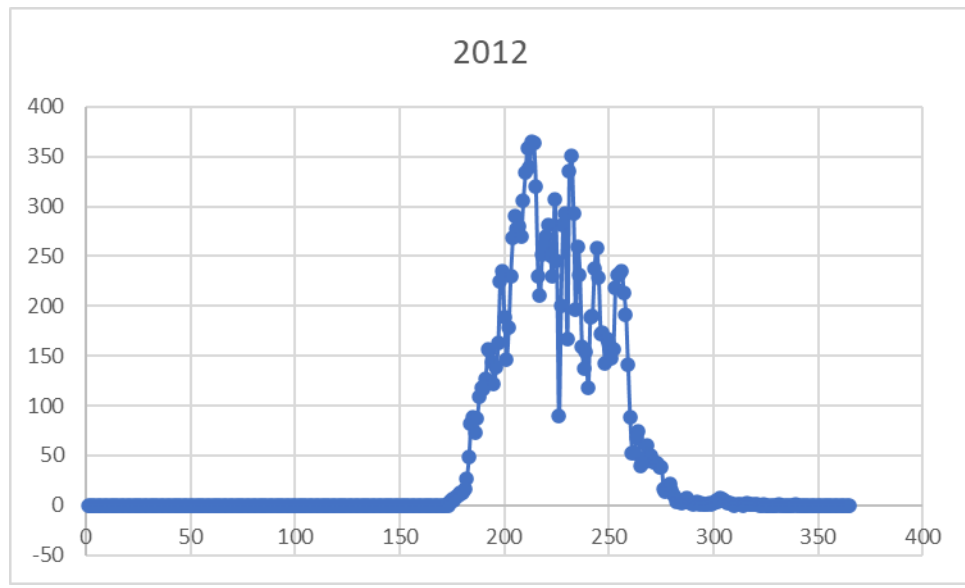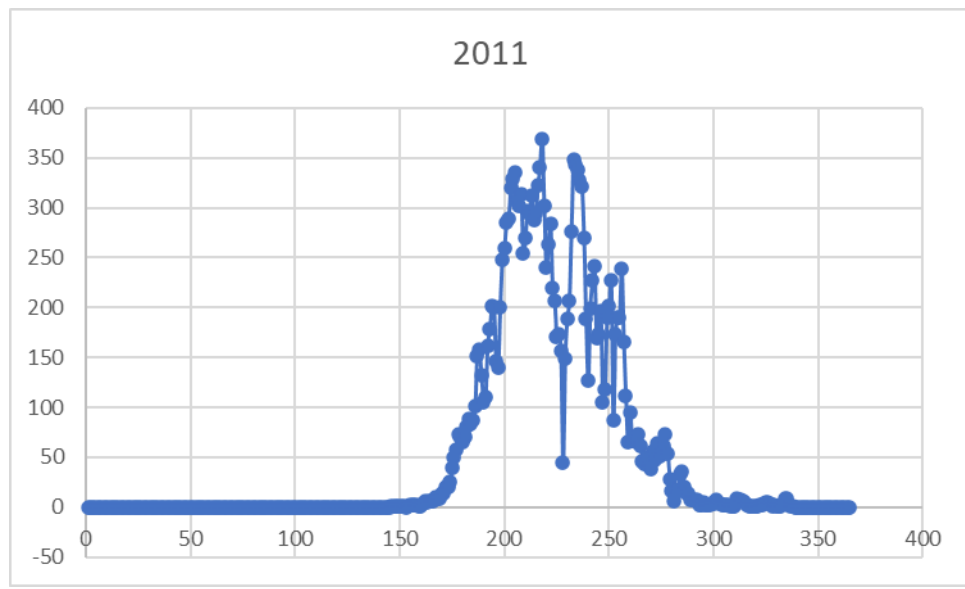

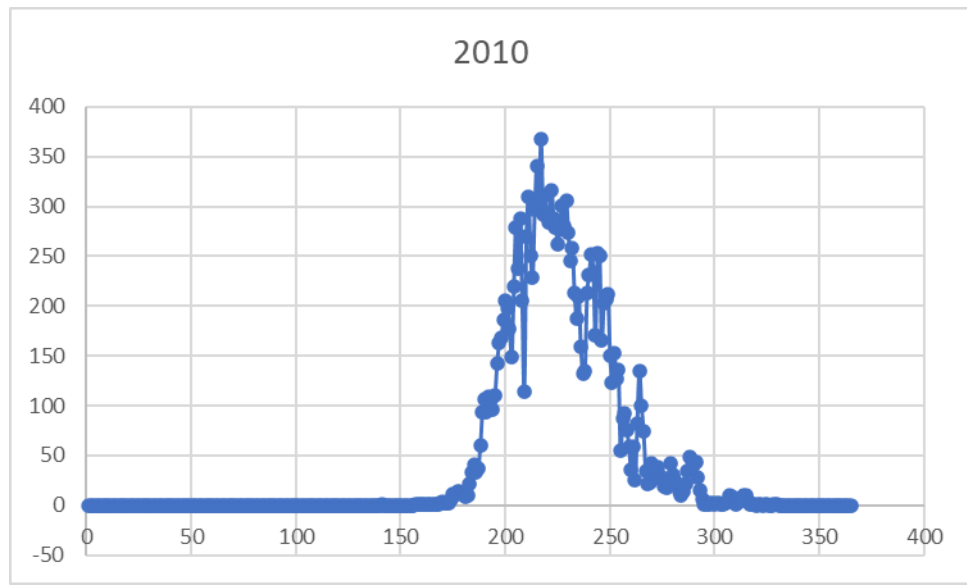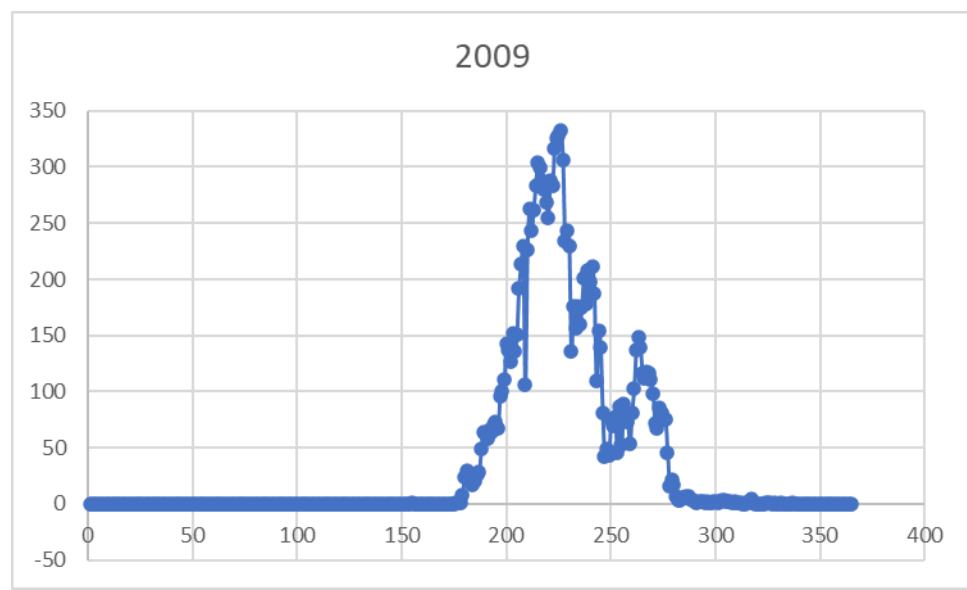

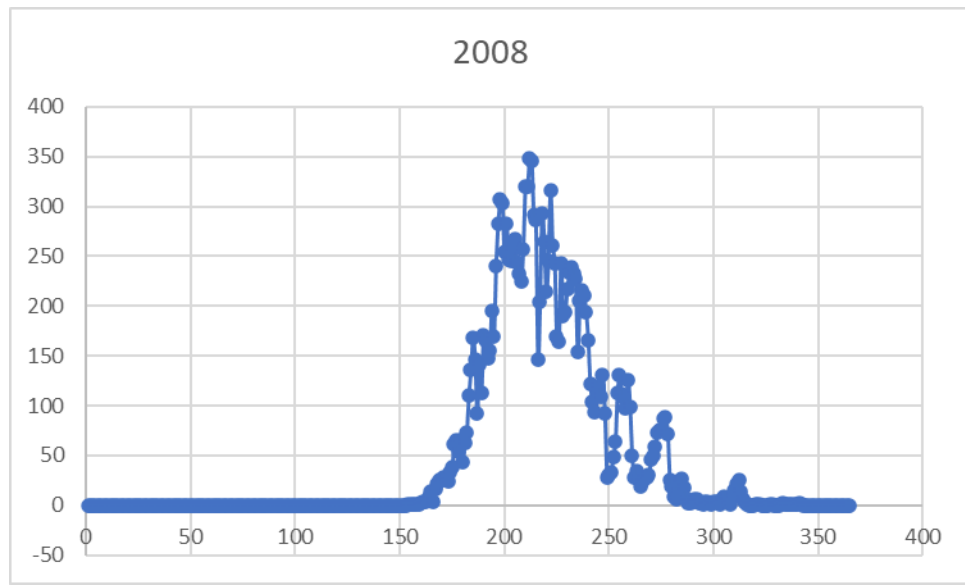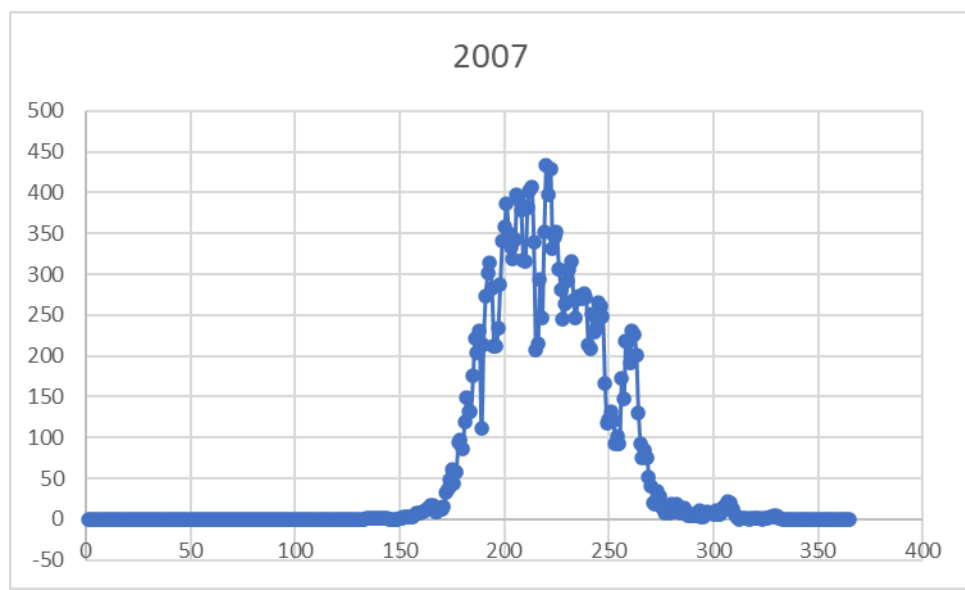

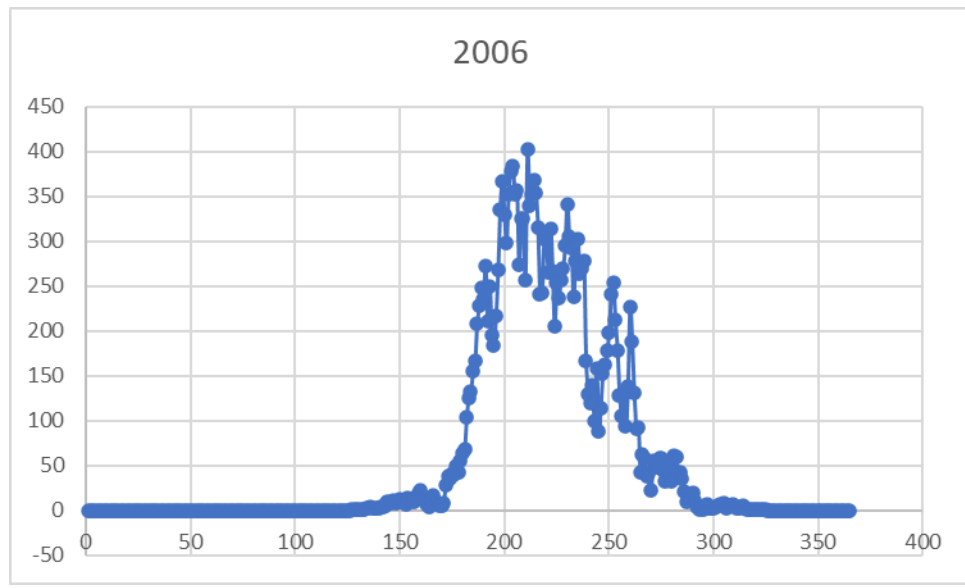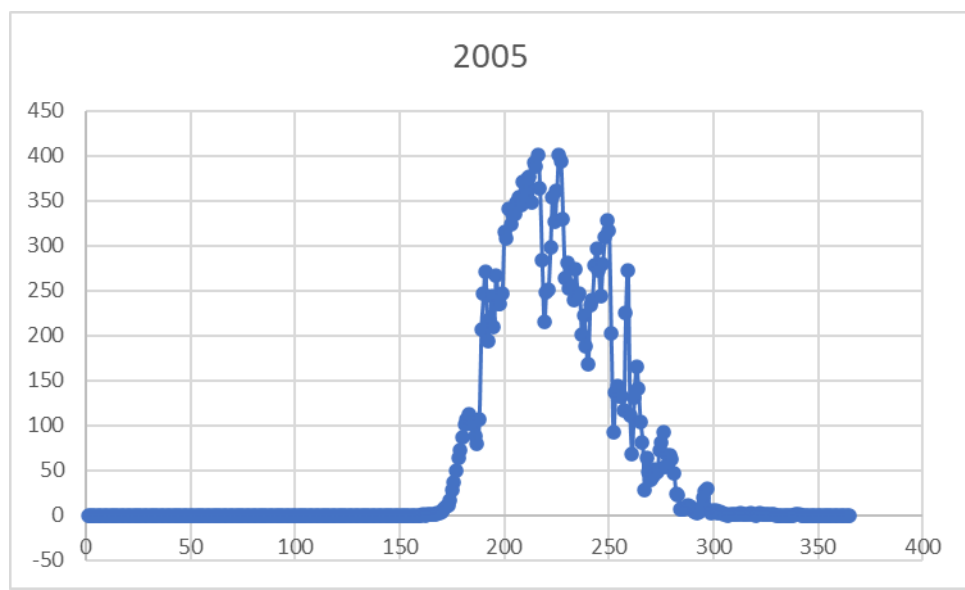

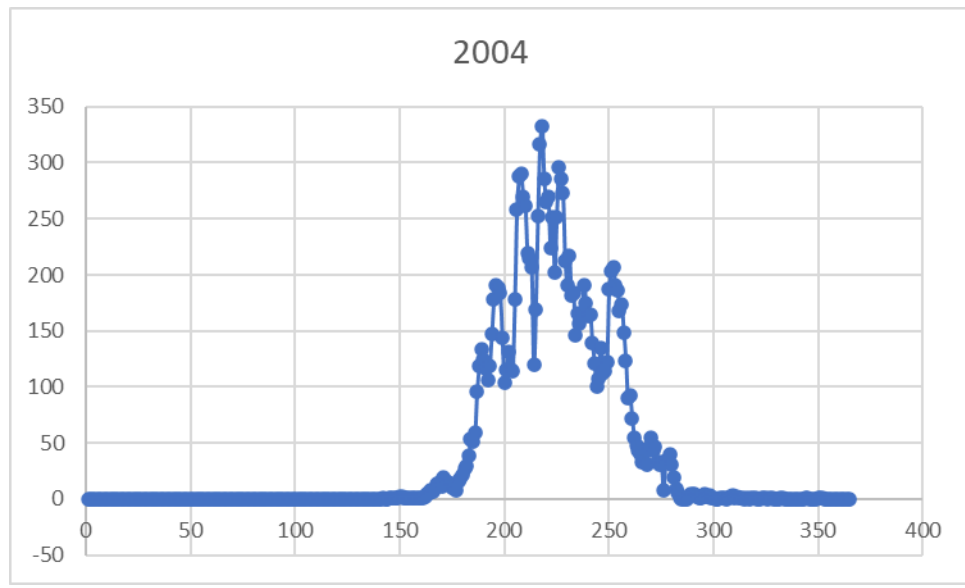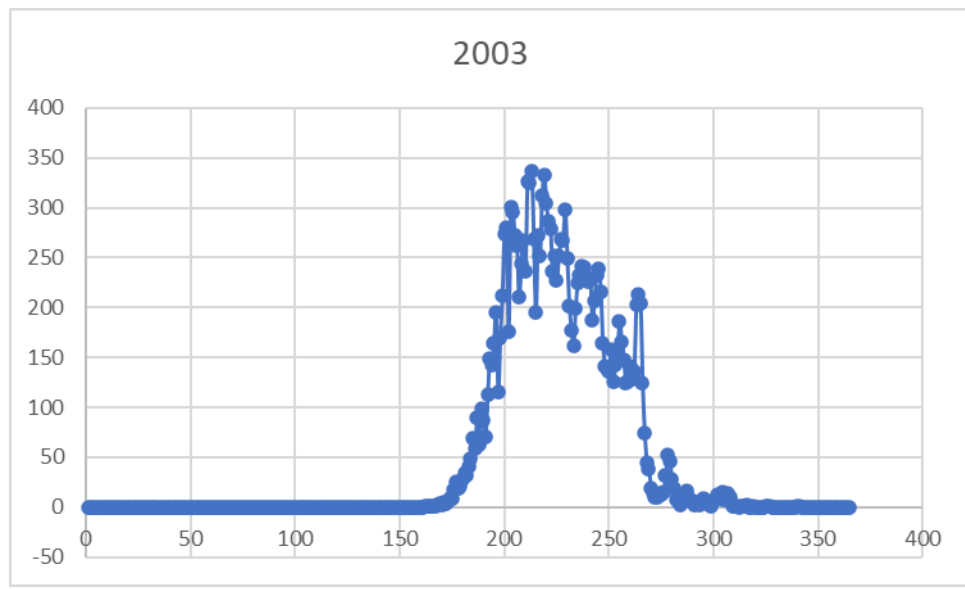

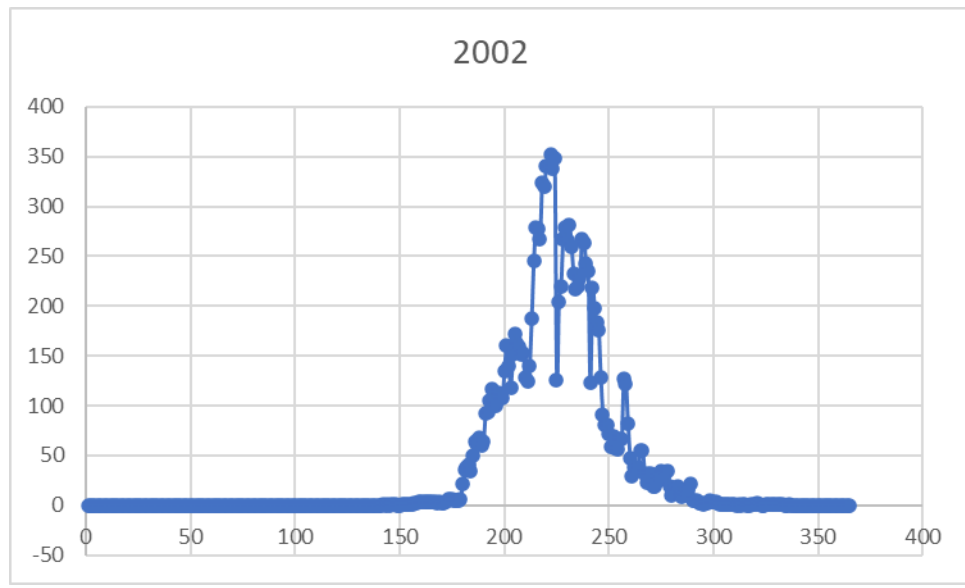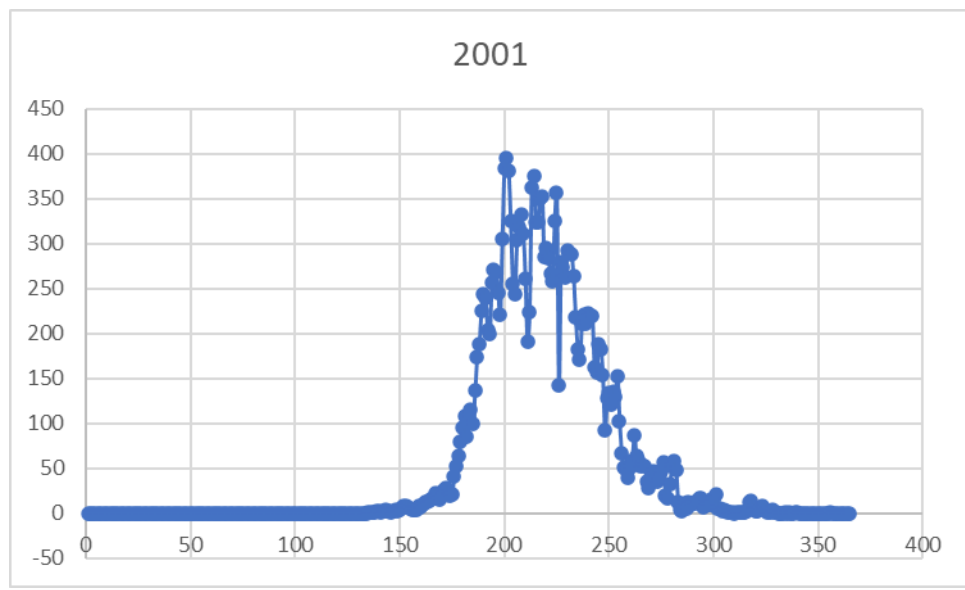

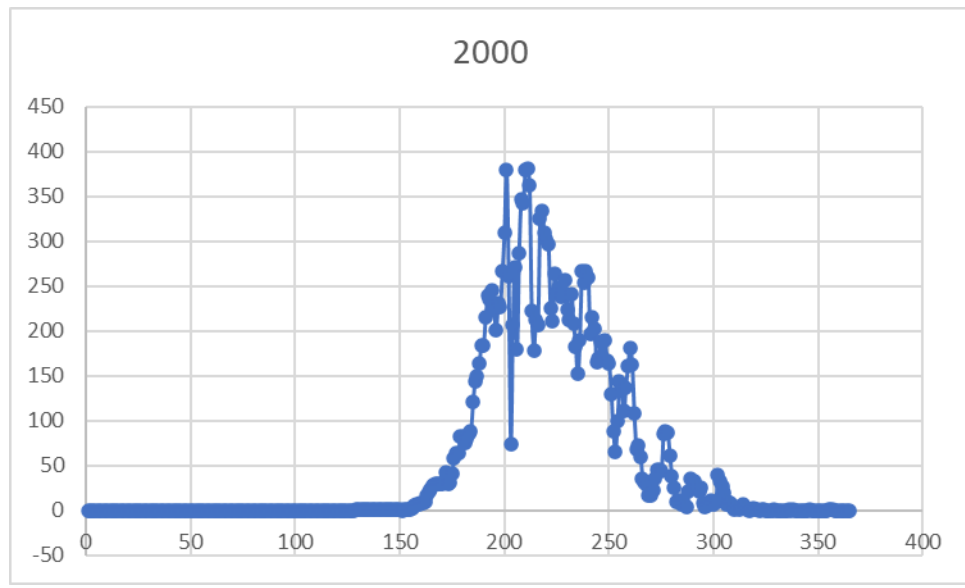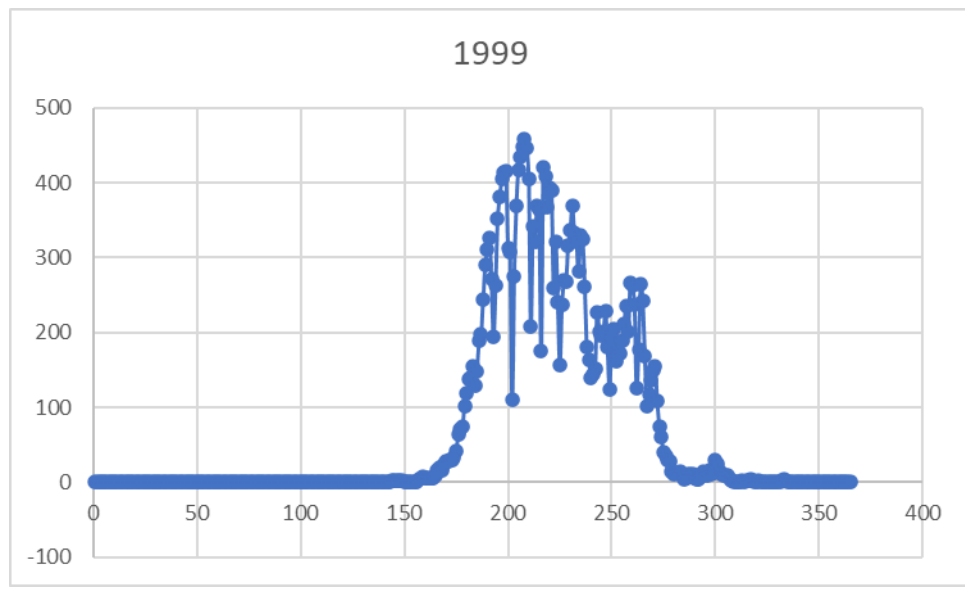

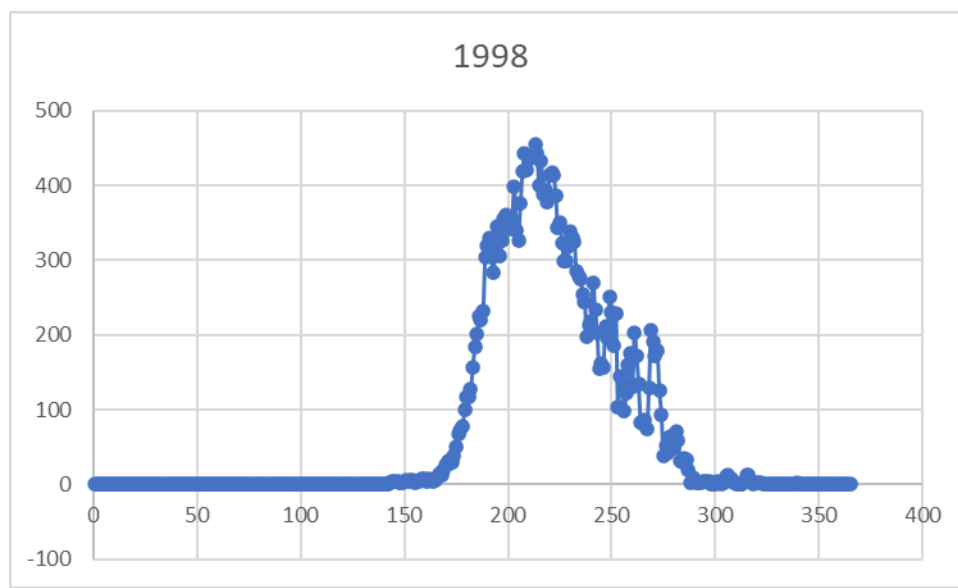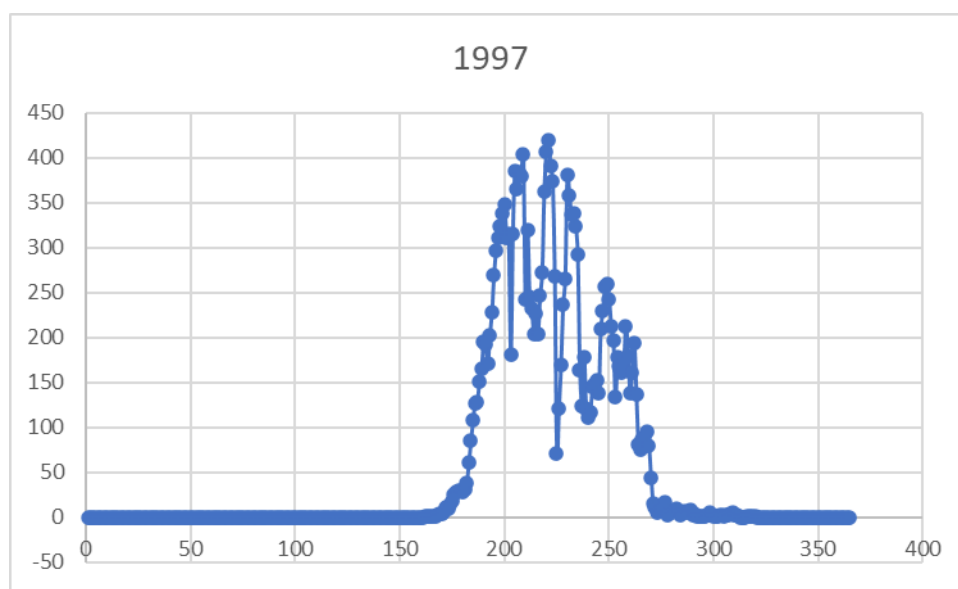

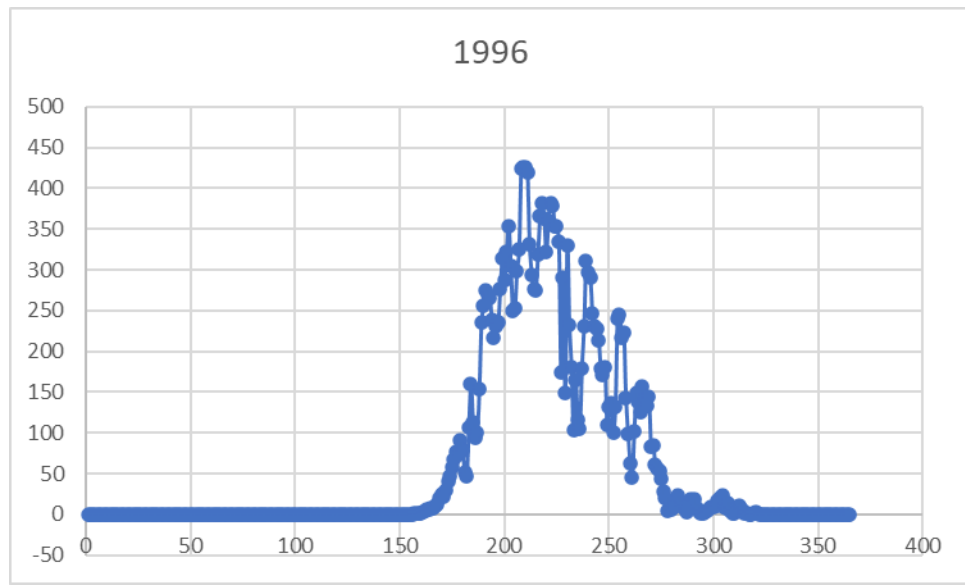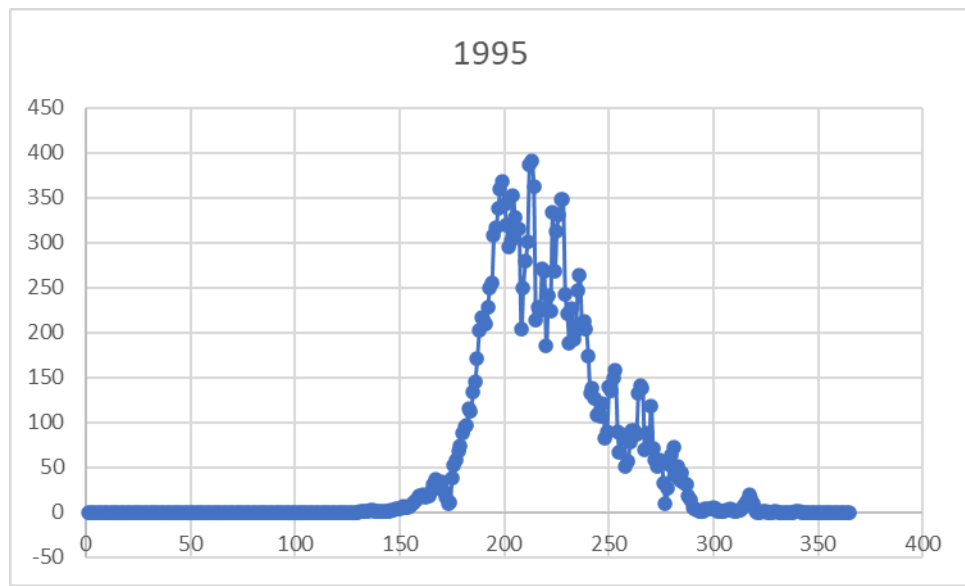

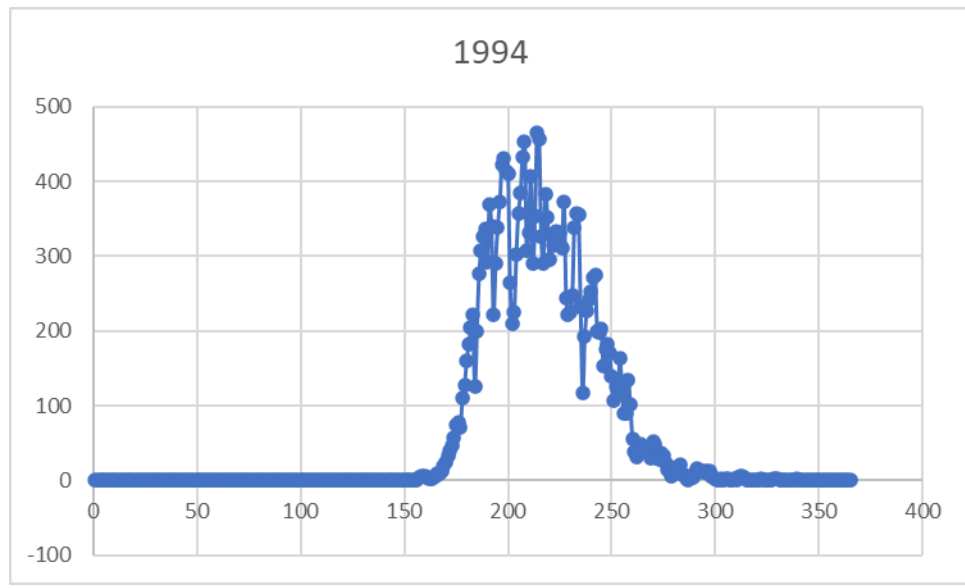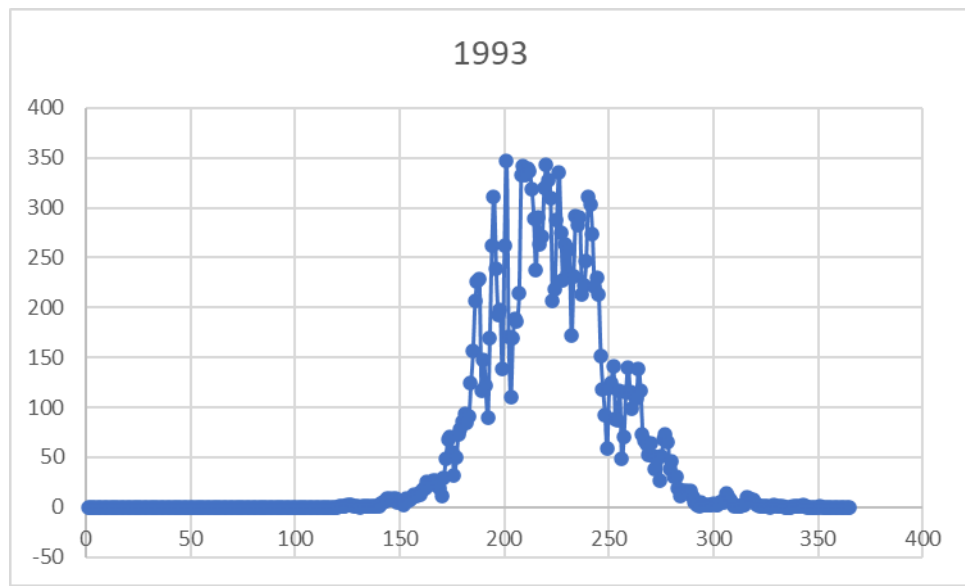

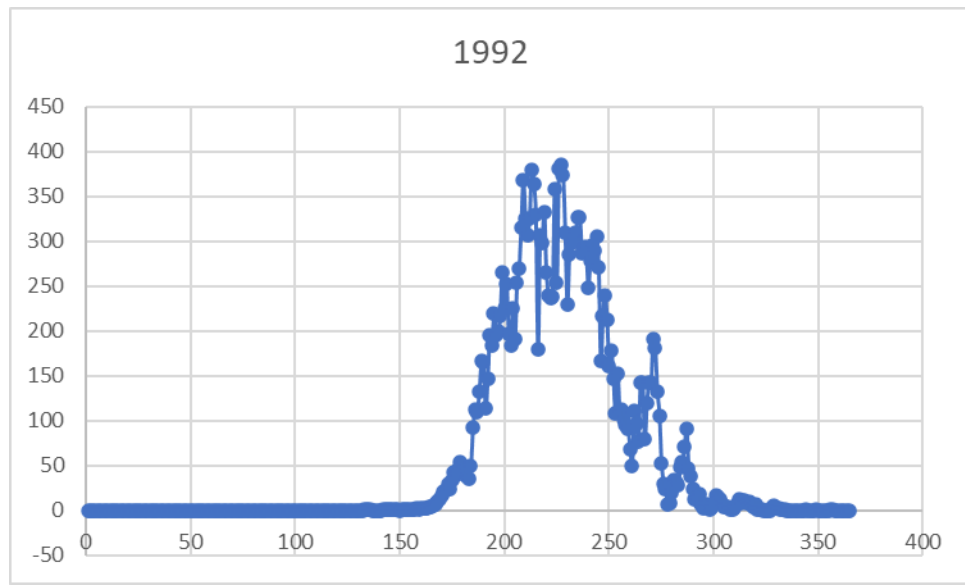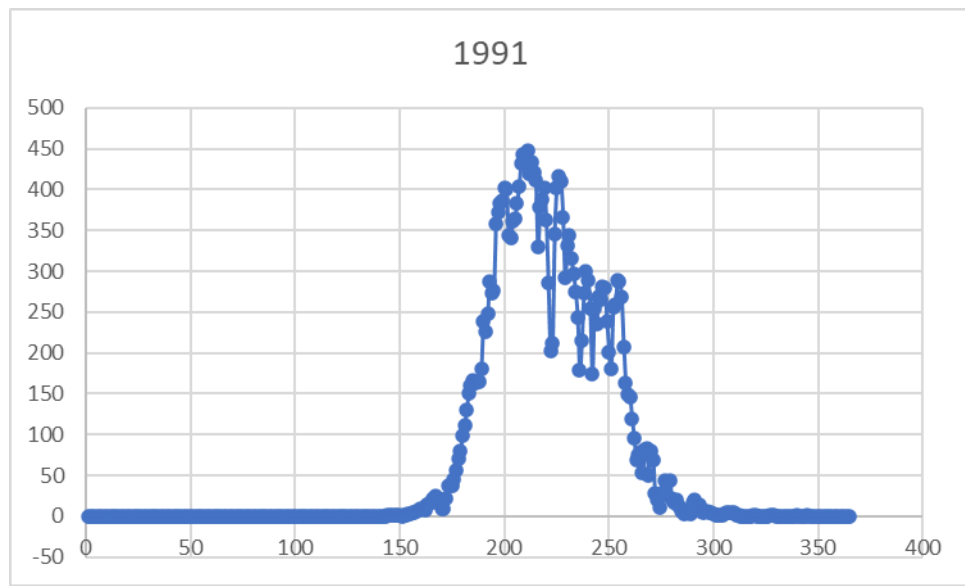

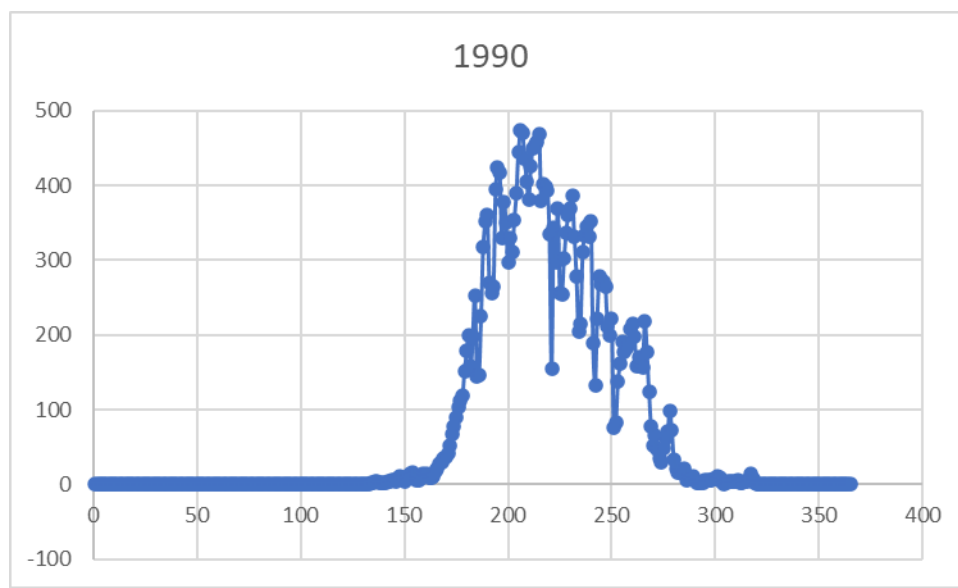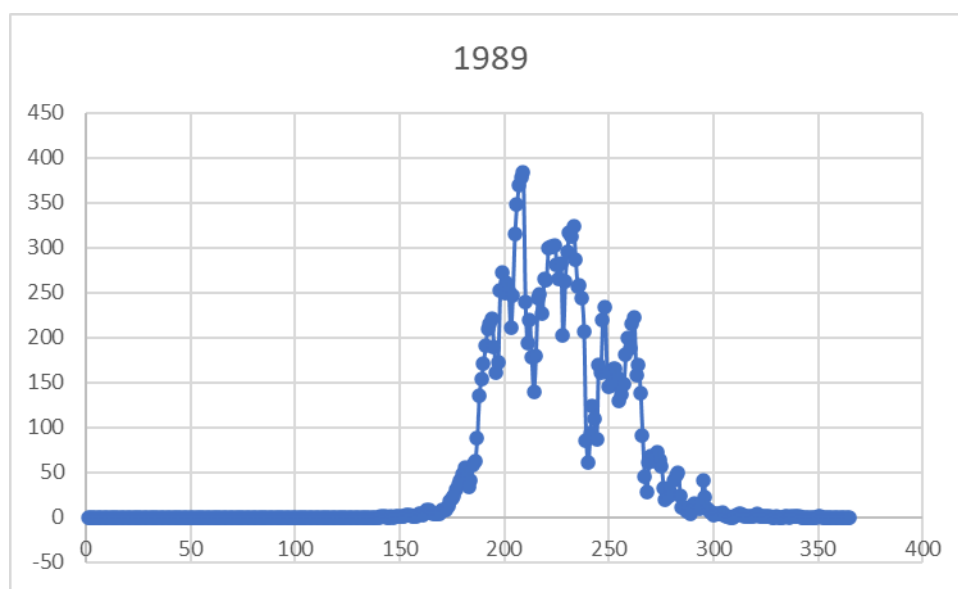

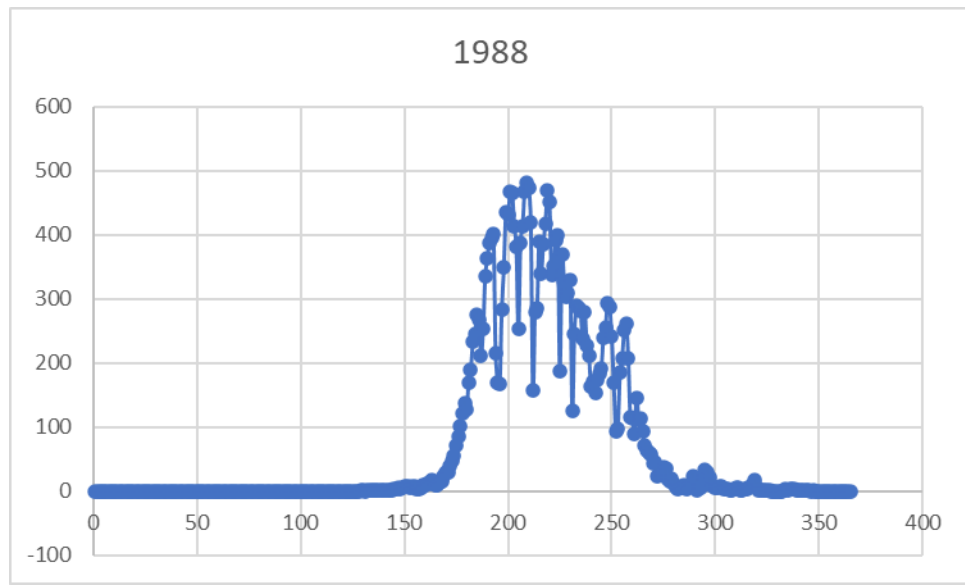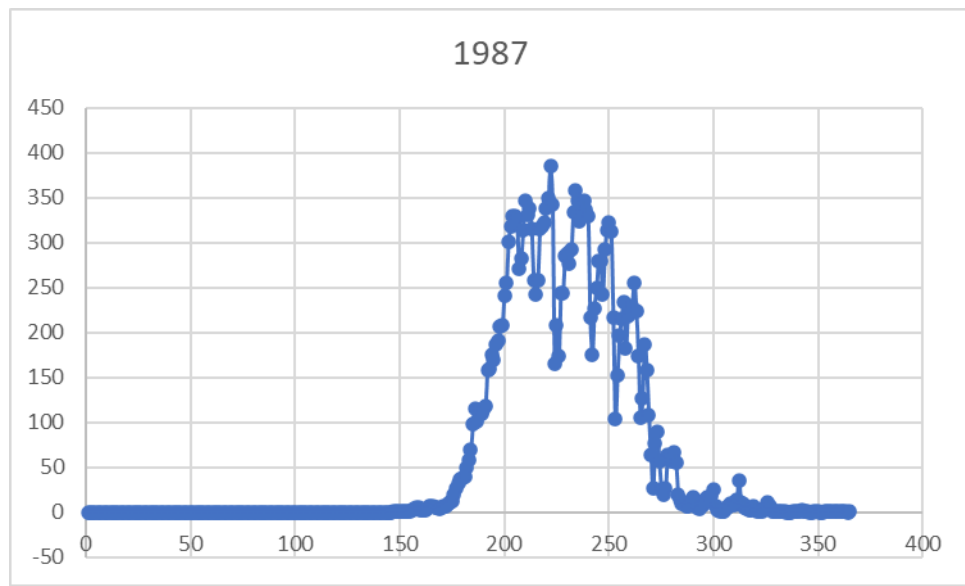

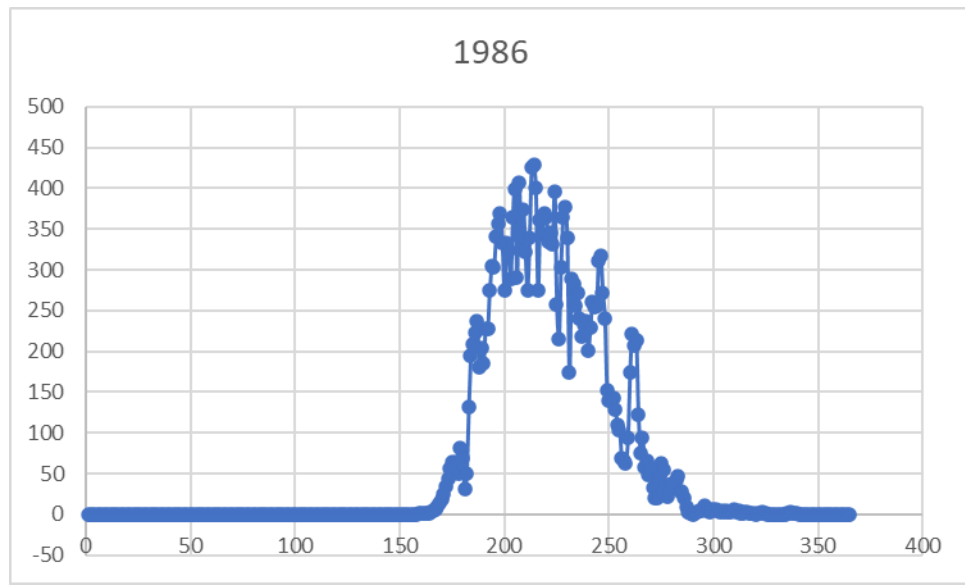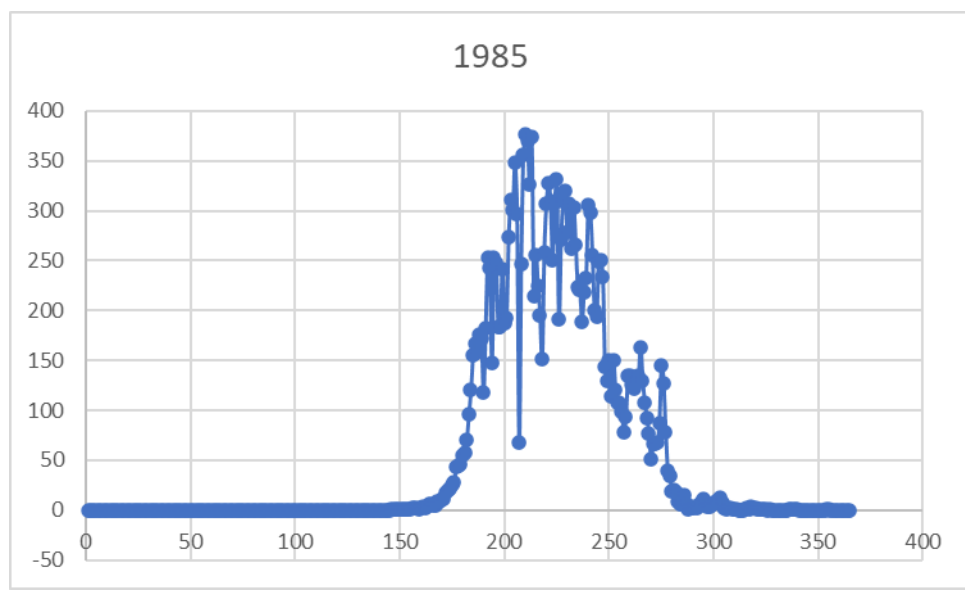

Supplement: Supplementary file 4 [file Data_Sheet_4.PDF]
